# Long-Term Aesthetic Performance of Freehand Direct Anterior Composite Resin Restorations: A Systematic Review and Meta-Analysis of Colour Alteration, Marginal Discolouration, and Surface-Quality Deterioration

**DOI:** 10.3390/dj14100633

**Published:** 2026-10-02

**Authors:** Luis Chauca-Bajaña, Gabriela Guadalupe Zambrano Manzaba, Luis Fernando Campos Lascano, Nayely Jimena Teran Sanchez, Andrea Ordoñez Balladares, Marcela Alejandra Ordoñez Balladares, Byron Velasquez Ron, Míriam Teulé-Trull, José Martín-Cruces

**Affiliations:** 1 School of Dentistry, Universidad Bolivariana del Ecuador, Duran 092406, Ecuador; 2 Cátedra Hispanoamericana de Investigación en Implantología Clínica y Prótesis Dental, Facultad de Ciencias de la Salud, Universidad Católica de Santiago de Guayaquil (UCSG)–SOADCO, Guayaquil 090101, Ecuador; 3 School of Dentistry, Universidad Católica de Santiago de Guayaquil (UCSG), Guayaquil 090101, Ecuador; 4 School of Dentistry, University of Guayaquil, Guayaquil 090101, Ecuador; 5 Carrera de Odontología, Universidad de Las Américas (UDLA), Quito 170102, Ecuador; byron.velasquez@udla.edu.ec; 6 Department of Surgery and Medical-Surgical Specialties, School of Medicine and Dentistry, University of Santiago de Compostela, 15701 Santiago de Compostela, Spain; 7 Department of Endodontics, Faculty of Dentistry, Universitat Internacional de Catalunya, 08195 Barcelona, Spain

**Keywords:** composite resin veneers, direct veneers, colour alteration, marginal discolouration, surface quality, meta-analysis

## Abstract

**Background**: Freehand direct composite resin veneers offer a minimally invasive approach for managing aesthetic alterations in anterior teeth; however, their long-term colour alteration and surface performance remain uncertain. This systematic review evaluated colour alteration, marginal discolouration, and surface-quality deterioration after at least 24 months of clinical service. **Methods**: MEDLINE/PubMed, Embase, Web of Science, Scopus, the Cochrane Library, and ClinicalTrials.gov were searched. Randomised and non-randomised trials and longitudinal clinical studies evaluating freehand direct anterior composite restorations were eligible. Risk of bias was assessed using RoB 2 and the JBI cohort checklist. Single-arm proportions were pooled separately using random-effects models with Freeman–Tukey transformation. Logit-REML models, binomial-normal generalised linear mixed models, Hartung–Knapp adjustments, leave-one-out analyses, and influence diagnostics were used as sensitivity analyses. **Results**: Eight studies were included. Colour alteration was evaluated in 893 restorations, with a pooled proportion of 14% (95% CI: 5–26%; I^2^ = 95.0%). Marginal discolouration involved 566 restorations and yielded a pooled proportion of 21% (95% CI: 10–34%; I^2^ = 91.8%). Surface-quality deterioration was assessed in 896 restorations, with a pooled proportion of 21% (95% CI: 9–35%; I^2^ = 95.6%). GLMM estimates were 11%, 19%, and 18%, respectively. Hartung–Knapp and leave-one-out analyses produced compatible results, although prediction intervals were wide. No study materially altered the overall interpretation. **Conclusions**: The pooled proportions of recorded aesthetic deterioration were 14% for colour alteration, 21% for marginal discolouration, and 21% for surface-quality deterioration. However, between-study heterogeneity was substantial, and prediction intervals were wide (0–63%, 0–68%, and 0–74%, respectively), indicating considerable variability in the outcomes expected across clinical settings. Absence of recorded deterioration should not be interpreted as equivalent to overall aesthetic success or clinical acceptability. Accordingly, the pooled estimates should be interpreted cautiously and primarily as average estimates across heterogeneous clinical settings.

## 1. Introduction

The appearance of the anterior teeth plays an important role in facial expression, self-confidence, and social interaction. Consequently, patients increasingly seek restorative treatments that can improve tooth colour, shape, proportion, and alignment while preserving as much sound dental tissue as possible. Within this context, direct composite resin restorations have evolved from a primarily reparative procedure into a versatile aesthetic treatment capable of producing clinically acceptable outcomes in the anterior region [1,2,3]. Their conservative nature, reparability, relatively low cost, and potential for completion in a single appointment make them particularly attractive when compared with more invasive indirect restorative alternatives.

Freehand direct composite resin veneers represent one of the most technique-sensitive applications of contemporary adhesive dentistry. In this approach, the clinician builds the facial surface directly on the tooth, reproducing dentinal opacity, enamel translucency, incisal effects, surface texture, and anatomical contours without relying on a laboratory-fabricated restoration. This flexibility allows treatment to be tailored to the individual patient and frequently requires little or no tooth preparation. However, the clinical result depends not only on the restorative material and adhesive system, but also on case selection, substrate quality, operator experience, incremental layering, polymerisation, finishing, and polishing procedures [4,5]. Unlike ceramic veneers, whose morphology and surface finish are largely established outside the mouth, freehand composite veneers depend heavily on the clinician’s ability to create and maintain an optically and biologically harmonious surface.

Available clinical evidence suggests that direct composite veneers can provide satisfactory medium- and long-term outcomes, although their performance varies across clinical situations. Direct veneers placed on vital teeth have demonstrated better performance than those placed on non-vital substrates, while existing restorations, occlusal loading, restorative complexity, and patient-related factors may also influence longevity [6,7,8]. Four-year evaluations of direct composite recontouring and diastema closure have reported favourable clinical behaviour and the possibility of extending service life through repair rather than complete replacement [9]. Earlier prospective observations similarly showed that direct composite additions can maintain acceptable aesthetic qualities over five years, although gradual changes in surface appearance may occur [10]. More recent investigations of template-assisted and prefabricated composite veneer techniques further demonstrate the growing range of direct veneering approaches, but their findings cannot necessarily be extrapolated to restorations constructed entirely freehand [11].

Survival alone, however, does not fully capture the clinical value of an anterior aesthetic restoration. A veneer can remain physically intact and still become unacceptable to the patient because its colour no longer matches the neighbouring teeth, its margins have darkened, or its surface has lost the texture and gloss that made the initial restoration appear natural. In long-term studies of anterior composites, aesthetic deterioration is consistently recognised as an important reason for intervention or replacement [1,12]. A 10-year practice-based comparison found that direct composite veneers generally exhibited lower longevity than ceramic veneers, underscoring the importance of examining how these restorations age rather than focusing exclusively on whether they remain in situ [5]. Although a recent systematic review and meta-analysis reported moderately high survival for resin composite laminate veneers, the available evidence combined direct and indirect approaches and primarily addressed overall survival and complications rather than the longitudinal behaviour of specific aesthetic domains [6].

Colour alteration is governed by a complex interaction between material composition and clinical exposure. Water sorption, incomplete polymer conversion, resin-matrix degradation, filler characteristics, surface irregularities, dietary chromogens, smoking, and oral hygiene may all contribute to intrinsic or extrinsic discolouration. Moreover, “colour alteration” is not measured uniformly. Some clinical studies use ordinal visual ratings derived from modified United States Public Health Service or FDI criteria, whereas instrumental studies may report changes through CIELAB or CIEDE2000 colour-difference equations. These metrics are related but not interchangeable. CIEDE2000 generally corresponds more closely to human visual perception than the traditional CIELAB formula, particularly because it accounts for differences in lightness, chroma, and hue [13]. Consequently, synthesis of this evidence requires careful separation or harmonisation of visual and instrumental outcomes.

Surface quality is closely connected to both colour alteration and marginal appearance. An initially smooth surface reflects light more uniformly, retains gloss, and is less prone to plaque and pigment accumulation. In contrast, progressive matrix wear, filler exposure, polishing defects, or loss of surface integrity can produce roughness and facilitate staining. Laboratory evidence shows that polishing performance varies across composite materials and that no single polishing system produces equivalent results for every composite formulation [14,15]. Exposure to staining solutions may further amplify differences in surface roughness and colour change [16]. Nevertheless, these laboratory findings cannot reproduce the full complexity of the oral environment, where mechanical loading, toothbrushing, dietary habits, salivary exposure, biofilm, and periodic professional maintenance act simultaneously over several years.

Marginal discolouration constitutes another clinically relevant but conceptually distinct outcome. It may reflect superficial pigment accumulation, marginal roughness, adhesive-interface degradation, or the early loss of marginal integrity. Importantly, marginal staining does not necessarily indicate secondary caries or structural failure, but it can compromise the perceived quality of a veneer and prompt polishing, repair, or replacement. In a randomized clinical trial of freehand direct laminate veneers, surface roughness and marginal discolouration were the principal qualitative deteriorations observed during follow-up [8]. Long-term investigations of anterior composites likewise indicate that material-related differences may become more apparent only after extended clinical service [17]. These observations support the need to evaluate aesthetic deterioration as a longitudinal process rather than as a single binary event.

Interpretation of the existing literature is complicated by considerable clinical and methodological heterogeneity. Studies differ in restorative indications, composite formulations, adhesive strategies, vitality of the treated teeth, operator experience, follow-up duration, evaluation criteria, and the thresholds used to define clinically acceptable performance [1,2,3,18]. The development of modern adhesive systems has substantially improved the predictability of direct bonded restorations, yet degradation of the adhesive interface and patient-level factors remain relevant over time [12,19]. Furthermore, comparisons between composite and ceramic veneers suggest that material selection involves a balance between tissue preservation, reparability, cost, aesthetic maintenance, and long-term durability rather than a simple choice between success and failure [5,20].

Despite the widespread clinical use of freehand direct anterior composite resin restorations, their long-term aesthetic behaviour remains insufficiently characterized. Previous systematic reviews have focused predominantly on survival, fracture, retention, and overall failure, often combining different restorative techniques. Consequently, less attention has been given to aesthetic deterioration occurring while restorations remain functional, particularly changes in colour match, marginal appearance, surface texture, and surface lustre. Evidence synthesis is further complicated by differences among modified USPHS, Ryge, and FDI evaluation systems, which do not use equivalent categories or thresholds. A structured synthesis based on predefined and harmonized definitions of aesthetic deterioration is therefore needed to obtain clinically interpretable estimates while acknowledging the diversity of the available evidence.

Accordingly, this systematic review and meta-analysis aimed to estimate the long-term proportions of colour alteration, marginal discolouration, and surface-quality deterioration in freehand direct anterior composite resin restorations after at least 24 months of clinical service. Eligible interventions included direct composite veneers, facial additions, composite build-ups, diastema-closure restorations, tooth reshaping, and aesthetic recontouring performed using a manual freehand technique. Secondary objectives were to evaluate the robustness of the pooled estimates across alternative statistical models, assess the influence of individual studies, and identify the principal methodological limitations affecting the interpretation and applicability of the available evidence.

## 2. Materials and Methods

**Figure 1:**
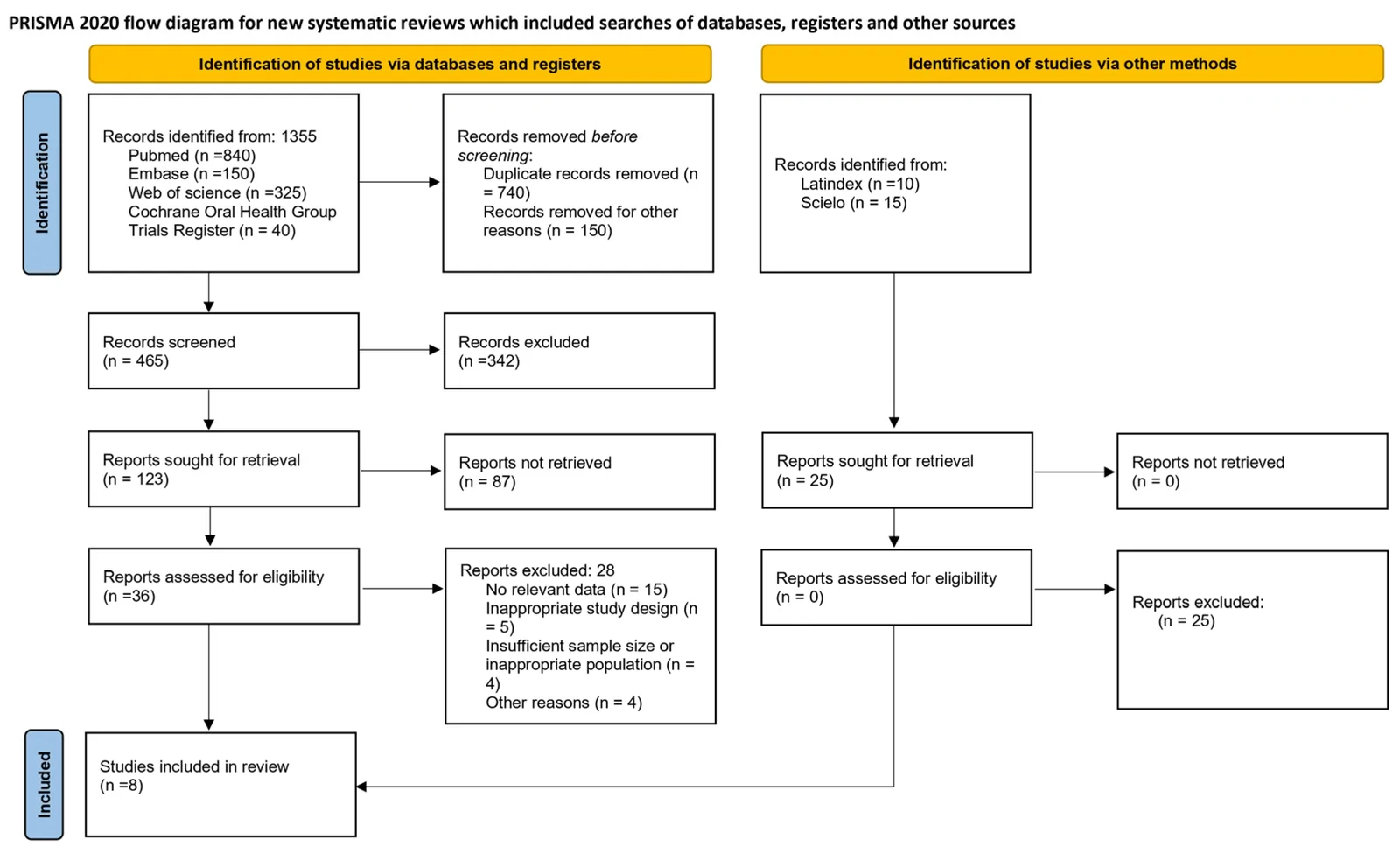
Flowchart of study selection.

A comprehensive search of the PROSPERO database was conducted in July 2026 to ensure that no similar ongoing reviews on the long-term aesthetic performance of freehand direct anterior composite resin restorations were registered. Subsequently, the protocol of this systematic review and meta-analysis was prospectively registered under the identification number CRD420261468096. The study was designed, conducted, and reported in accordance with the PRISMA 2020 statement, and the corresponding flow diagram is presented in Figure 1. In addition, the completed PRISMA 2020 checklist is provided as Supplementary Material (Table S1) [21].

### 2.1. PICO Question

Among adolescent and adult patients receiving freehand direct anterior composite resin restorations, what is their long-term aesthetic performance regarding colour alteration, marginal discolouration, and surface quality after at least 24 months of clinical service?

#### PICO Framework

Population (P): Adolescent and adult patients receiving freehand direct composite resin restorations on permanent anterior teeth for aesthetic or minimally invasive restorative indications, including tooth discolouration, morphological abnormalities, diastema, minor malposition, enamel defects, tooth reshaping, aesthetic recontouring, or replacement of unaesthetic pre-existing restorations.

Although differences may exist in patient characteristics, clinical indications, restorative extension, composite materials, evaluation criteria, and follow-up periods, all included studies must clinically evaluate freehand direct anterior composite resin restorations and report at least one outcome related to colour alteration, marginal discolouration, or surface quality. Intervention (I): Freehand direct anterior composite resin restorations placed intraorally on permanent anterior teeth, including direct composite veneers, diastema closure restorations, composite build-ups, facial composite additions, tooth reshaping, and aesthetic recontouring.

Comparison (C): Baseline aesthetic condition of the same restorations, an earlier follow-up assessment, or another eligible composite resin material, layering approach, finishing and polishing protocol, or clinical subgroup. Single-arm longitudinal studies without an independent control group will also be eligible when they report extractable aesthetic outcomes over time.

Outcomes (O):

Primary outcomes:

- Colour alteration or colour match—assessed visually using validated clinical criteria or instrumentally through colour-difference measurements, including ΔE*ab or ΔE**_00_**.
- Marginal discolouration or marginal staining—assessed using FDI, modified USPHS/Ryge, or other validated clinical criteria.
- Surface quality—including surface texture, surface roughness, gloss retention, or loss of surface lustre.

Secondary outcomes:

- Overall aesthetic acceptability.
- Anatomical form and surface morphology.
- Patient-reported aesthetic satisfaction.
- Need for repolishing, refurbishment, or repair due to aesthetic deterioration.
- Veneer replacement or aesthetic failure.
- Overall survival and success of the restorations.

### 2.2. Search Strategy and Database Screening

The study selection process was conducted using the Rayyan platform (Qatar Computing Research Institute, Doha, Qatar), which facilitated the identification, organization, deduplication, and blinded screening of the retrieved records.

A comprehensive and systematic literature search was carried out across multiple electronic databases, including MEDLINE via PubMed, EMBASE via Ovid, Web of Science, Scopus, the Cochrane Library, and ClinicalTrials.gov. Additionally, five regional databases of the World Health Organization—AIM, LILACS, IMEMR, IMSEAR, and WPRIM—were explored to ensure broad geographical coverage of the available evidence. To minimize publication bias and identify the relevant grey literature, conference proceedings were also screened through the Conference Proceedings Citation Index.

The search strategy was customized for each database using a combination of controlled vocabulary terms, including Medical Subject Headings (MeSH) and Emtree terms, and free-text keywords related to direct composite resin veneers and their long-term aesthetic performance. The principal search terms included “dental veneers”, “direct veneers”, “direct composite veneers”, “composite resin veneers”, “resin composite”, “freehand technique”, “direct layering”, “anterior teeth”, “colour alteration”, “color match”, “colour change”, “marginal discolouration”, “marginal staining”, “surface quality”, “surface texture”, “surface roughness”, “surface gloss”, “clinical performance”, “aesthetic failure”, and “long-term follow-up”. Boolean operators (AND/OR) were systematically applied to combine search terms and optimize both the sensitivity and specificity of the search. The final electronic searches were conducted in June 2026. The search strategy was adapted to the syntax and indexing system of each database, using the same core concepts related to direct composite veneers, anterior restorations, colour alteration, marginal discolouration, surface quality, and long-term clinical performance. Controlled vocabulary terms and free-text keywords were combined using the Boolean operators AND and OR, with database-specific modifications where required. No restrictions on publication date were applied.

To ensure completeness, a manual search of the reference lists of eligible studies and previously published systematic or narrative reviews was performed to identify additional potentially relevant articles that might not have been retrieved through the electronic search. All identified records were imported into the Rayyan platform, where duplicate entries were detected and removed before screening. Subsequently, titles and abstracts were independently evaluated by two reviewers to determine their eligibility according to the predefined inclusion and exclusion criteria. Potentially eligible reports were retrieved for full-text assessment.

### 2.3. Eligibility Criteria

Inclusion criteria:

1. Study design: Randomized and non-randomized clinical trials, prospective and retrospective cohort studies, longitudinal observational studies, and clinical case series providing sufficient quantitative data for analysis.
2. Population: Adolescent or adult patients receiving freehand direct composite resin restorations on permanent anterior teeth for aesthetic or minimally invasive restorative indications, including tooth discolouration, morphological abnormalities, diastema, minor malposition, enamel defects, tooth reshaping, aesthetic recontouring, or replacement of unaesthetic pre-existing restorations.
3. Intervention: Freehand direct anterior composite resin restorations constructed intraorally through manual composite placement, sculpting, and layering. Eligible interventions included direct composite veneers, facial composite additions, direct composite build-ups, diastema closure restorations, tooth reshaping, and aesthetic recontouring. No laboratory fabrication, prefabricated veneer system, injectable composite technique, clear template, or digitally guided placement technique was permitted.
4. Comparison: The baseline aesthetic condition of the restoration, an earlier eligible follow-up assessment, another composite resin material, a different enamel preparation or adhesive protocol, an alternative layering approach, a finishing and polishing protocol, or another eligible clinical subgroup. Single-arm longitudinal studies without an independent comparator were also eligible when they provided extractable aesthetic outcome data over time.
5. Follow-up: A minimum clinical follow-up of 24 months was required. When a study reported multiple eligible follow-up assessments, data from the longest available follow-up were used in the primary quantitative synthesis. Earlier assessments were recorded during data extraction but were not entered simultaneously into the same meta-analysis, thereby avoiding statistical dependency and double counting of restorations from the same clinical cohort.
6. Outcomes: Studies were required to report at least one quantitative or clinically classifiable outcome related to colour alteration or colour match, marginal discolouration or marginal staining, surface texture, surface roughness, surface gloss, loss of surface lustre, or overall aesthetic quality. Outcomes assessed using FDI, modified USPHS/Ryge, or other validated clinical criteria were eligible. Instrumental colour measurements based on CIELAB or CIEDE2000 systems, including ΔE*ab and ΔE**_00_**, were also considered eligible.
7. Data reporting: Studies were required to report the number of patients and restorations evaluated, the duration of clinical follow-up, and sufficient outcome data for extraction or conversion. Eligible formats included means and standard deviations, event counts, proportions, frequencies, ordinal clinical ratings, or other convertible quantitative measures. When attrition occurred, the number of restorations clinically evaluated at each follow-up assessment had to be identifiable.
8. Language: Full-text articles published in English were eligible.
9. Publication type: Peer-reviewed original clinical research articles were included.

Exclusion criteria:

Studies were excluded when they met any of the following conditions:

1. Evaluated indirect composite or ceramic restorations without a separately reported freehand direct anterior composite resin group.
2. Evaluated prefabricated composite veneers, injectable composite techniques, clear-template techniques, index-guided restorations, or digitally guided techniques without separately extractable data for a freehand direct composite group.
3. Evaluated conventional Class III or Class IV restorations placed primarily for the treatment of caries or traumatic fractures without an aesthetic tooth-reshaping, facial veneering, recontouring, or diastema-closure component.
4. Evaluated posterior composite restorations, full-coverage composite crowns, indirect restorations, palatal veneers without facial aesthetic reconstruction, or restorations placed exclusively for the management of tooth wear without separately extractable anterior aesthetic data.
5. Had a clinical follow-up shorter than 24 months.
6. Reported exclusively survival, fracture, retention, debonding, postoperative sensitivity, secondary caries, periodontal response, or other biological or functional outcomes without at least one eligible aesthetic outcome.
7. Were exclusively in vitro or laboratory investigations of colour alteration, staining susceptibility, polishing, roughness, gloss, wear, water storage, thermocycling, or artificial ageing.
8. Were case reports, small descriptive clinical reports without sufficient quantitative data, narrative reviews, systematic reviews, meta-analyses, editorials, letters, technical reports, expert opinions, or conference abstracts without adequate outcome information.
9. Did not report quantitative or clinically classifiable data related to colour, marginal discolouration, or surface quality.
10. Combined direct and indirect techniques, freehand and guided techniques, anterior and posterior restorations, or different restorative indications without providing separately extractable data for the eligible freehand direct anterior composite restorations.
11. Reported overlapping patient populations or successive follow-ups of the same clinical cohort. When multiple publications originated from the same cohort, they were linked and treated as a single study. The publication with the longest eligible follow-up was prioritized, while companion reports were used to obtain additional non-duplicated outcomes or earlier follow-up measurements.

Although variability was anticipated in composite resin composition, adhesive strategy, clinical indication, enamel preparation, restorative extension, layering procedure, finishing and polishing protocol, assessment system, and follow-up duration, studies were considered eligible when they investigated comparable aesthetic outcomes in freehand direct anterior composite resin restorations after at least 24 months of clinical service. Eligible restorative approaches included direct facial veneers, composite additions, build-ups, diastema closures, tooth reshaping, and aesthetic recontouring; however, the specific restorative indication and extension were recorded for subgroup and sensitivity analyses.

To address the expected clinical and methodological heterogeneity, a random-effects meta-analytic approach was used. When multiple eligible treatment groups were reported within the same study, event counts and denominators were combined to obtain a single study-level estimate for each aesthetic outcome, thereby avoiding the treatment of correlated observations as independent. When multiple eligible follow-up assessments were available, the longest follow-up was selected for the primary quantitative synthesis. Effect measures were selected according to the nature of the reported data, including pooled proportions for single-arm categorical outcomes, risk ratios or odds ratios for comparative dichotomous outcomes, mean differences when identical continuous scales were used, and standardized mean differences when conceptually comparable outcomes were reported using different measurement systems. Full facial composite veneers and other anterior aesthetic composite restorations were additionally planned to be examined in separate subgroups or sensitivity analyses whenever sufficient studies were available.

### 2.4. Study Selection Process and Data Extraction

Two investigators (CC and AO) independently conducted study selection and data extraction using a standardized and pilot-tested extraction form developed specifically for this review. Disagreements were resolved through discussion and, when consensus could not be reached, by consultation with a third investigator (B.V.R.). Before formal extraction, the reviewers independently piloted the form using a sample of eligible studies to ensure consistency in the interpretation and classification of the aesthetic outcomes.

For each included study, the following information was extracted: first author, publication year, country, study design, participant characteristics, clinical indication, type of anterior restoration, composite resin and adhesive system, restorative technique, number of patients and restorations at baseline and final follow-up, comparison groups, duration of follow-up, clinical assessment system, examiner calibration or blinding, and the principal findings. Outcome-specific data were collected for colour alteration or colour mismatch, marginal discolouration or staining, and deterioration of surface quality, including surface roughness, loss of lustre, and surface texture. For quantitative synthesis, the number of restorations evaluated and the number presenting each aesthetic event were extracted for every eligible study group and follow-up period. Where studies reported several clinical categories, outcome harmonization was performed within the original assessment system used in each study. Categories representing the ideal aesthetic condition were classified as non-events, whereas any category indicating deterioration from the ideal condition was classified as an event. FDI, modified USPHS, and modified Ryge categories were not considered directly equivalent across studies; rather, dichotomization was performed independently within each original assessment framework. Therefore, the resulting event estimates represent loss of ideal aesthetic status and should not be interpreted as clinical failure or clinical unacceptability. The study-level event counts, denominators, original assessment systems, and study-specific harmonization rules used for the quantitative synthesis are provided in Supplementary Table S2. The principal characteristics of the included studies are presented in Table 1.

The selection process was performed in two stages. First, the titles and abstracts of all retrieved records were independently screened against the predefined eligibility criteria. Records considered potentially relevant by either reviewer proceeded to full-text evaluation. Second, the complete reports were independently examined to confirm the study design, freehand direct composite technique, eligible anterior restorations, minimum follow-up of 24 months, and availability of at least one extractable aesthetic outcome. The reasons for excluding articles after full-text assessment were recorded and incorporated into the PRISMA 2020 flow diagram.

When studies reported multiple treatment groups or follow-up assessments, these data were initially extracted separately and identified according to study, treatment group, outcome, and assessment time. For the primary meta-analysis, eligible direct composite groups were combined when clinically appropriate, and only the longest eligible follow-up was retained. Consequently, each independent study contributed a maximum of one effect estimate to each outcome-specific meta-analysis, avoiding double counting and dependency among observations. Multiple publications originating from the same patient cohort were cross-checked using author names, clinical setting, recruitment dates, sample characteristics, restorative materials, and follow-up periods to prevent double counting. When several reports described the same cohort, the most complete dataset was used, while complementary non-duplicated information from related publications was incorporated when necessary.

When numerical information was available only in tables or figures, data were extracted directly from the original presentation and independently verified by both reviewers. If essential information remained unavailable or unclear, the corresponding author was contacted by email on up to three occasions, with a one-week interval between attempts. The final extracted dataset was cross-checked against the original articles before statistical analysis. The methodological and clinical characteristics of the included studies, together with their principal aesthetic findings, are summarized in Table 1.

### 2.5. Assessment of Risk of Bias (RoB) and Methodological Quality

The risk of bias and methodological quality of the included studies were independently evaluated by three reviewers using appraisal tools selected according to the design of each study. Randomized clinical trials were assessed using the revised Cochrane Risk of Bias tool (RoB 2) [28], whereas prospective and retrospective longitudinal cohort studies without a causal non-randomized intervention comparison were evaluated using the Joanna Briggs Institute (JBI) Critical Appraisal Checklist for Cohort Studies [29]. RoB 2 assessments were performed at the outcome level and considered bias arising from the randomization process, deviations from the intended interventions, missing outcome data, outcome measurement, and selection of the reported result. When domain-level judgements were identical for colour alteration, marginal discolouration, and surface-quality deterioration, a single consensus assessment was reported for the study.

The JBI cohort checklist was applied at the study level and examined participant-group comparability, validity and reliability of exposure and outcome measurements, identification and management of confounding factors, adequacy and completeness of follow-up, management of incomplete follow-up, and appropriateness of the statistical analysis. Individual JBI items were classified as “Yes”, “No”, “Unclear”, or “Not applicable”. No numerical quality score or post hoc classification into low, moderate, or high methodological quality was generated from the JBI responses, thereby preserving the domain-specific interpretation of each study.

Particular attention was given to examiner calibration, blinding of outcome assessors, completeness of long-term follow-up, handling of missing data, and the validity and reliability of the criteria used to evaluate colour alteration, marginal discolouration, and surface quality, as these aesthetic outcomes may be particularly susceptible to assessor subjectivity. Disagreements among reviewers were resolved through discussion and, when necessary, consultation with an additional investigator until consensus was reached. RoB 2 results were presented using traffic-light and unweighted summary plots, whereas JBI assessments were reported separately using item-level appraisal matrices and response-distribution charts.

### 2.6. Statistical Analysis

A quantitative synthesis was conducted to estimate the long-term occurrence of aesthetic deterioration in freehand direct anterior composite resin restorations. Three clinically distinct outcomes were analysed separately: colour alteration, marginal discolouration, and surface-quality deterioration. For each outcome, the number of restorations presenting deterioration and the total number of restorations evaluated at the final eligible follow-up were extracted from each study. The restoration was used as the unit of analysis because aesthetic outcomes were primarily reported at the restoration level. As individual patients could contribute more than one restoration, observations within studies may not have been fully independent. No additional adjustment for within-patient clustering was applied at the meta-analysis level because patient-level correlation data, intraclass correlation coefficients, or other parameters required for a consistent cluster-adjusted analysis were not sufficiently available across studies. Therefore, study-level event counts and denominators were analysed as reported in the original publications. For the primary quantitative synthesis, outcome harmonization was performed within the original assessment system used in each study. Categories representing the ideal aesthetic condition were classified as non-events, whereas any category indicating deterioration from the ideal condition was classified as an event. FDI, modified USPHS, and modified Ryge categories were not assumed to be directly equivalent across studies; rather, dichotomization was performed independently within each original assessment framework. Accordingly, an event represented loss of ideal aesthetic status and should not be interpreted as clinical failure or clinical unacceptability. When a study included more than one eligible direct composite group, event counts and denominators were combined to obtain a single study-level estimate so that each study contributed only one estimate per outcome and multiple treatment arms were not treated as independent study estimates. Data from indirect ceramic groups were not incorporated into the pooled proportions.

The primary analyses were conducted as single-arm meta-analyses of proportions. Study-specific proportions and their corresponding 95% confidence intervals (CIs) were calculated and pooled using a random-effects model to account for anticipated clinical and methodological heterogeneity. The Freeman–Tukey double-arcsine transformation was selected for the primary analysis because some included studies reported zero events and several study-specific proportions were close to the boundaries of the proportion scale, conditions under which variance instability may occur. This transformation allowed zero-event studies to be incorporated without the need for arbitrary continuity corrections. Pooled estimates were subsequently back-transformed and expressed as proportions to facilitate clinical interpretation. Because transformed-proportion meta-analysis has recognized limitations, particularly regarding the interpretation of back-transformed pooled estimates, the robustness of the findings was additionally evaluated using alternative modelling approaches, including logit-transformed random-effects models with REML estimation and binomial-normal generalized linear mixed models. Results were reported as the proportion of restorations showing deterioration from the ideal aesthetic category; the complementary proportion remaining in the ideal category was also calculated descriptively.

Between-study heterogeneity was assessed using Cochran’s Q test and quantified using the between-study variance (τ^2^) and I^2^ statistic. I^2^ values of approximately 25%, 50%, and 75% were interpreted as representing low, moderate, and high heterogeneity, respectively. A 95% prediction interval was calculated for each primary outcome to estimate the range of proportions that could be expected in a future clinically comparable study. Random-effects modelling was selected a priori because variation was expected in composite formulations, adhesive protocols, clinical indications, finishing and polishing procedures, assessment systems, and follow-up durations.

Several sensitivity analyses were conducted to evaluate the robustness of the primary findings. First, proportions were analysed using a logit-transformed random-effects model with between-study variances estimated by restricted maximum likelihood (REML). Second, a binomial-normal generalized linear mixed model (GLMM) with a logit link was fitted directly to the event counts and denominators, thereby avoiding the need for continuity corrections in studies with zero events. Third, the logit-REML model was recalculated using the Hartung–Knapp adjustment to provide more conservative confidence intervals in view of the limited number of studies and substantial heterogeneity. Agreement in the direction and magnitude of the estimates obtained from the Freeman–Tukey, logit-REML, GLMM, and Hartung–Knapp analyses was considered evidence of result robustness.

Leave-one-study-out analyses were performed for each aesthetic outcome by sequentially excluding one study and recalculating the pooled GLMM estimate. Influence and outlier diagnostics were additionally examined using externally studentized residuals, DFFITS, Cook’s distance, and hat values derived from the logit-transformed REML model. A study was considered potentially atypical or influential when one or more diagnostic values exceeded their conventional thresholds; however, decisions regarding study exclusion were based on the combined diagnostic evidence and the clinical and methodological characteristics of the study rather than on an isolated statistical criterion.

Potential small-study effects were explored through visual inspection of funnel plots. Because only eight studies were available for colour alteration and surface-quality deterioration and six for marginal discolouration, funnel plots were considered exploratory. Egger’s regression test and trim-and-fill procedures were not performed because fewer than 10 independent studies were available for each outcome. Similarly, subgroup analyses and meta-regressions were not undertaken because the number of studies was insufficient to generate reliable moderator estimates without substantial risk of overfitting.

A multilevel meta-analytic model was initially prespecified because several studies were expected to contribute multiple treatment groups or eligible follow-up assessments. However, treatment groups were combined when clinically appropriate and only the longest eligible follow-up was included in the final quantitative synthesis. Consequently, each study contributed a maximum of one independent estimate to each outcome-specific meta-analysis. A conventional random-effects model was therefore considered more appropriate than a multilevel model. This modification from the prespecified analytical approach was made to avoid double counting and unnecessary model complexity given the limited number of independent studies.

All statistical analyses were performed using R software version 4.4.1 (R Foundation for Statistical Computing, Vienna, Austria), principally with the meta and metafor packages. Statistical tests were two-sided, and *p*-values below 0.05 were considered statistically significant. Because of the anticipated heterogeneity and limited number of studies, clinical interpretation emphasized the magnitude and precision of the pooled proportions, their prediction intervals, and the consistency of the sensitivity analyses rather than statistical significance alone. Given the harmonization approach described above, pooled proportions were interpreted as estimates of deterioration from the ideal aesthetic condition rather than as rates of clinically unacceptable restoration or restorative failure.

## 3. Results

The RoB 2 assessment showed that four randomized clinical trials presented some concerns and one study was judged to have a high overall risk of bias. Outcome measurement was considered at low risk in all trials because validated clinical criteria and calibrated, blinded evaluators were generally used. The principal concerns involved incomplete long-term follow-up and the absence of prospectively available statistical analysis plans. The JBI appraisal of the retrospective cohort studies showed adequate outcome measurement, follow-up duration, and statistical analysis. However, limitations were identified regarding participant comparability, baseline outcome documentation, control of confounding, and management of incomplete follow-up. All three cohort studies were retained for the systematic review and meta-analysis (Table 1, Table 2 and Table 3).

### 3.1. Pooled Proportion of Colour Alteration

Eight independent studies comprising 893 freehand direct anterior composite resin restorations were included in the quantitative synthesis of colour alteration. Using a random-effects model with Freeman–Tukey double-arcsine transformation, the pooled proportion of restorations presenting colour alteration was 14% (95% CI: 5–26%). Equivalently, approximately 86% of the restorations were free from recorded colour alteration at the final eligible assessment, indicating generally favourable long-term colour performance. Individual study estimates ranged from 0% to 35%, and the relatively balanced distribution of study weights indicated that no single study disproportionately influenced the overall estimate.

**Figure 2:**
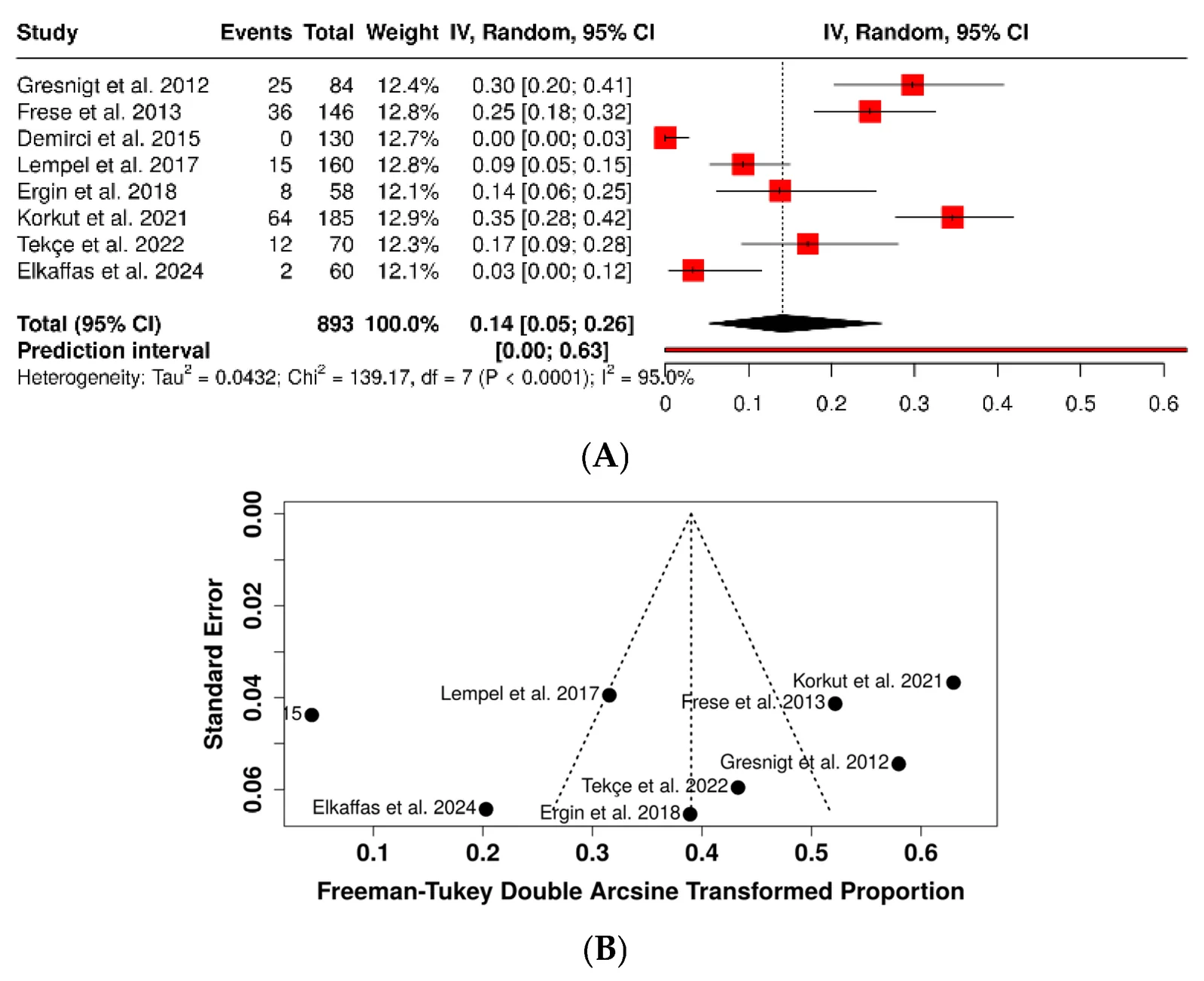
Meta-analysis of colour alteration in freehand direct anterior composite resin restorations. (**A**) Forest plot showing the pooled proportion of colour alteration obtained using a random-effects model with Freeman–Tukey double-arcsine transformation. (**B**) Funnel plot illustrating the distribution of study estimates and potential small-study effects [8,9,22,23,24,25,26,27].

Considerable between-study heterogeneity was observed (τ^2^ = 0.0432; Cochran’s Q = 139.17, df = 7, *p* < 0.0001; I^2^ = 95.0%). Furthermore, the 95% prediction interval ranged from 0% to 63%, indicating that the proportion of colour alteration observed in a future comparable study could vary substantially. Thus, although the pooled findings were clinically encouraging, colour performance was not uniform across studies, potentially reflecting differences in restorative materials, clinical indications, operative procedures, assessment systems, patient characteristics, and follow-up duration. Visual inspection of the funnel plot showed considerable dispersion and an apparently asymmetric distribution of the study estimates around the pooled effect. Several estimates were located outside the pseudo-95% confidence limits, particularly those corresponding to very low or relatively high proportions of colour alteration. However, this pattern should be interpreted cautiously because only eight studies were included and substantial heterogeneity was present. The observed asymmetry may therefore reflect genuine clinical and methodological differences rather than publication bias or small-study effects. Consequently, the funnel plot was considered exploratory, and publication bias could neither be confirmed nor excluded (Figure 2).

### 3.2. Sensitivity Analysis of Colour Alteration

**Figure 3:**
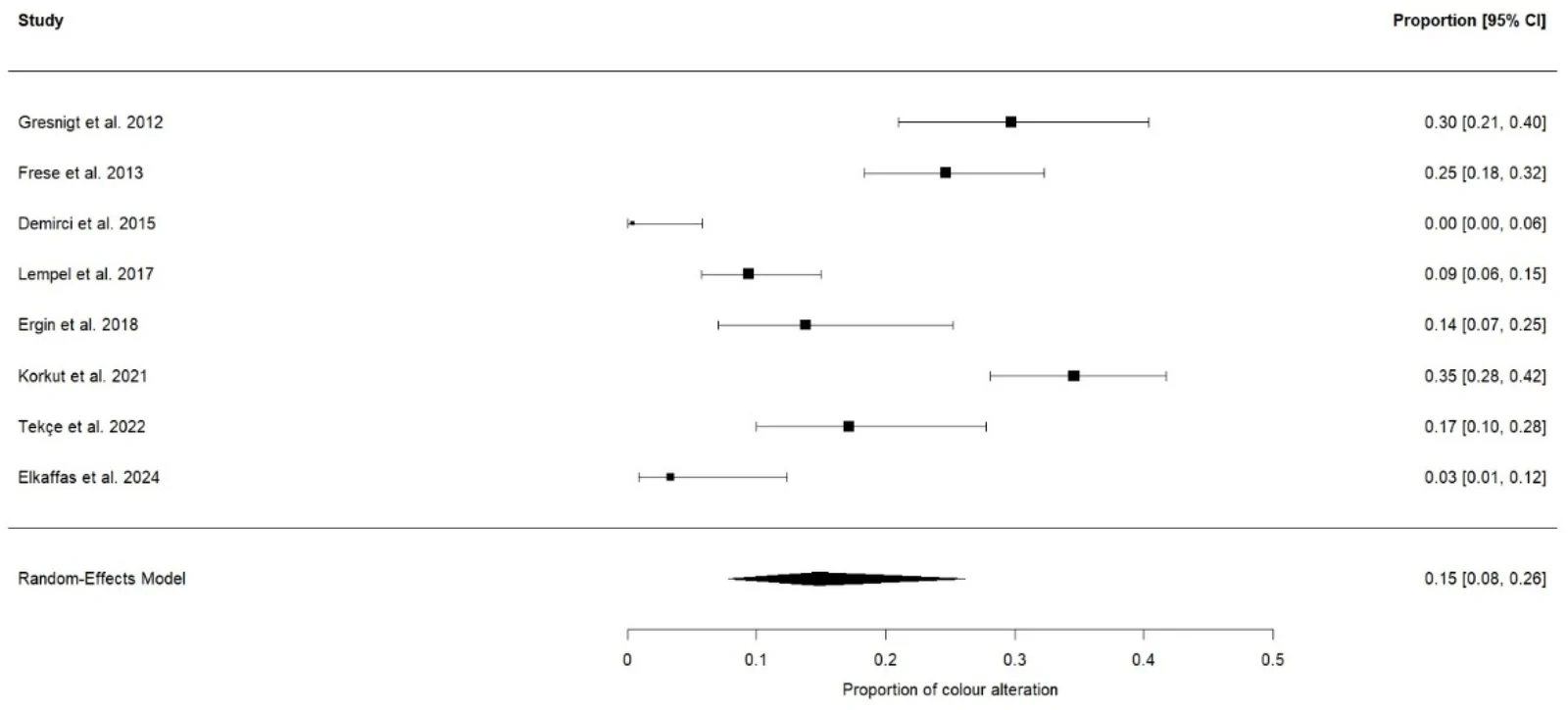
Forest plot of the pooled proportion of colour alteration in freehand direct anterior composite resin restorations using a logit-transformed random-effects model with REML estimation [8,9,22,23,24,25,26,27].

Eight independent studies comprising 893 freehand direct anterior composite resin restorations were included in the sensitivity analysis. Using a random-effects model based on logit-transformed proportions and restricted maximum likelihood estimation, the pooled proportion of restorations presenting colour alteration was 15% (95% CI: 8–26%). Accordingly, approximately 85% of the restorations were free from recorded colour alteration at the final eligible assessment, supporting generally favourable long-term colour performance. Individual study estimates ranged from 0% to 35%, demonstrating variability in colour outcomes across clinical settings. Importantly, this estimate was consistent with the primary Freeman–Tukey analysis, which yielded a pooled proportion of 14% (95% CI: 5–26%). The close agreement between both analytical approaches supports the robustness of the overall finding (Figure 3).

### 3.3. GLMM Analysis of Colour Alteration

**Figure 4:**
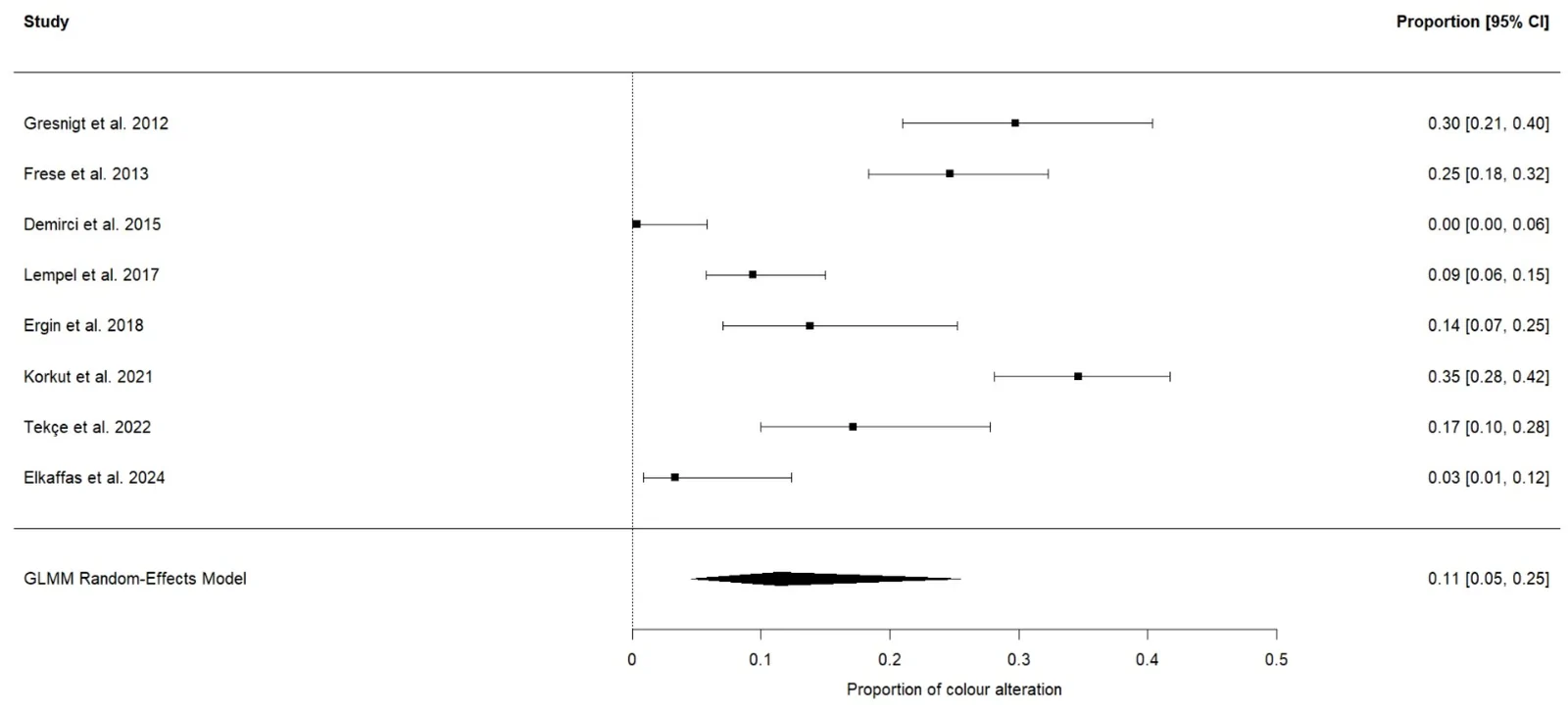
Forest plot of the pooled proportion of colour alteration in freehand direct anterior composite resin restorations using a binomial-normal generalized linear mixed model [8,9,22,23,24,25,26,27].

Eight independent studies comprising 893 freehand direct anterior composite resin restorations were included in the generalized linear mixed-model analysis. The binomial-normal GLMM estimated a pooled proportion of colour alteration of 11% (95% CI: 5–25%). Equivalently, approximately 89% of the restorations were free from recorded colour alteration at the final eligible assessment, indicating favourable long-term colour performance. Individual study estimates ranged from 0% to 35%, demonstrating variability in colour outcomes across the included clinical settings. The GLMM estimate was slightly lower than those obtained using the Freeman–Tukey model (14%; 95% CI: 5–26%) and the logit-REML model (15%; 95% CI: 8–26%). Nevertheless, the confidence intervals overlapped substantially, and all three approaches consistently indicated that colour alteration affected a minority of restorations, supporting the robustness of the overall conclusion (Figure 4).

### 3.4. Hartung–Knapp Sensitivity Analysis of Colour Alteration

**Figure 5:**
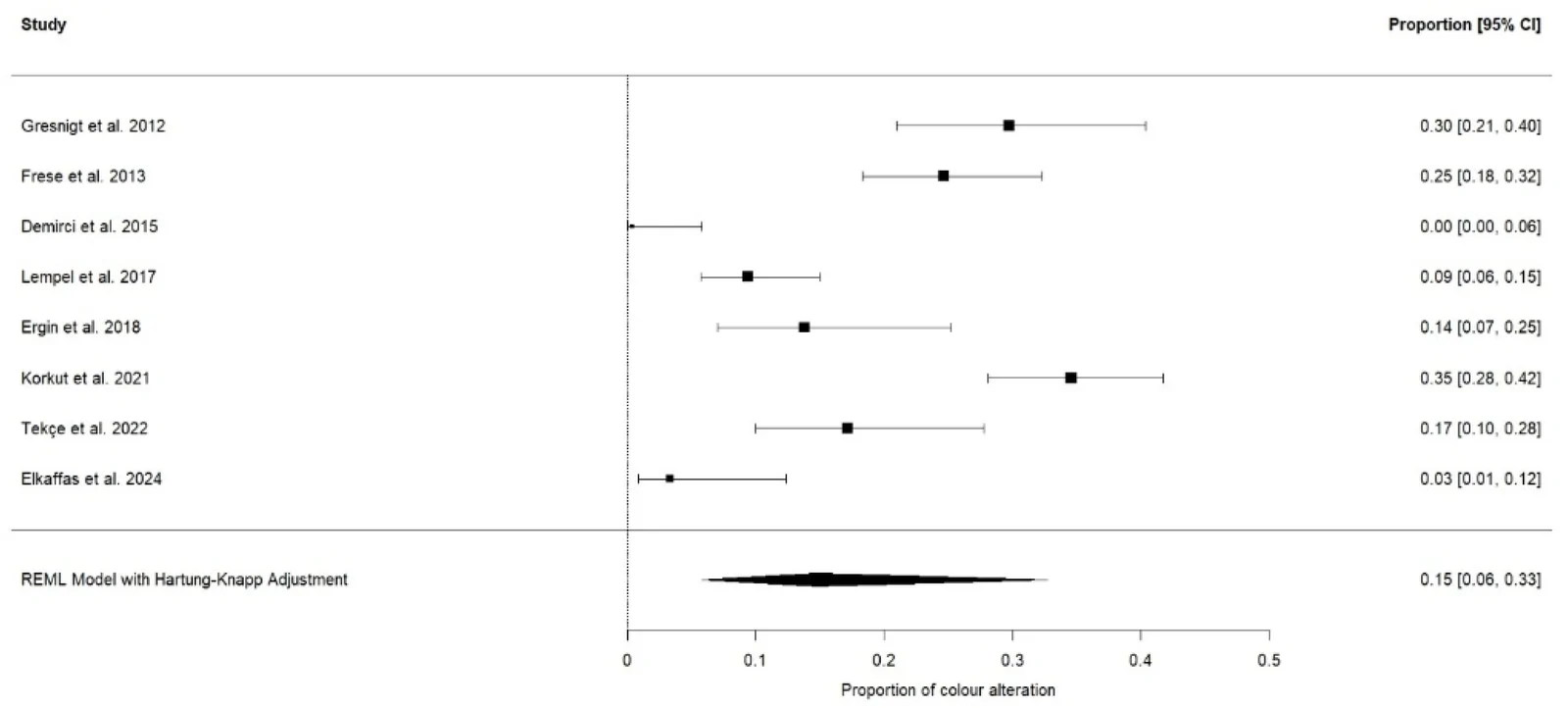
Forest plot of the pooled proportion of colour alteration in freehand direct anterior composite resin restorations using a REML random-effects model with Hartung–Knapp adjustment [8,9,22,23,24,25,26,27].

Eight independent studies comprising 893 freehand direct anterior composite resin restorations were included in the Hartung–Knapp sensitivity analysis. The REML random-effects model with Hartung–Knapp adjustment yielded a pooled colour-alteration proportion of 15% (95% CI: 6–33%). Accordingly, approximately 85% of the restorations were free from recorded colour alteration at the final eligible assessment, supporting generally favourable long-term colour performance. The pooled estimate remained unchanged from the conventional logit-REML model; however, the confidence interval widened from 8–26% to 6–33%, reflecting the additional uncertainty associated with the limited number of studies and the estimation of between-study heterogeneity. The Hartung–Knapp estimate was also compatible with the GLMM result of 11% (95% CI: 5–25%), as their confidence intervals overlapped substantially. Overall, the sensitivity analysis supported the direction of the primary finding while indicating greater uncertainty regarding its precise magnitude (Figure 5).

### 3.5. Leave-One-Out Sensitivity Analysis of Colour Alteration

**Figure 6:**
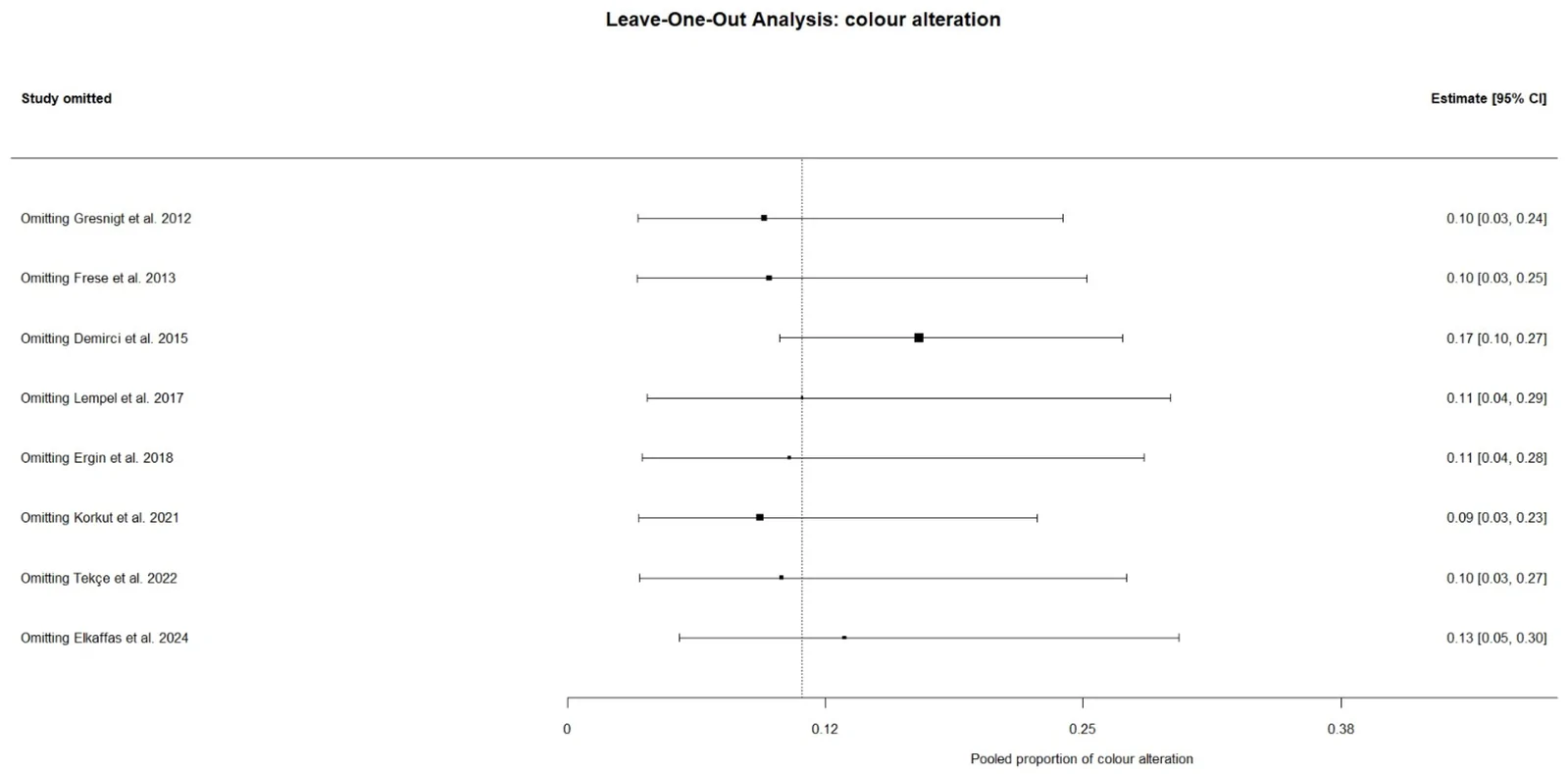
Leave-one-out sensitivity analysis of the pooled proportion of colour alteration in freehand direct anterior composite resin restorations. The vertical dotted line represents the estimate obtained from the complete GLMM [8,9,22,23,24,25,26,27].

The leave-one-out sensitivity analysis demonstrated that the pooled proportion of colour alteration was generally robust to the sequential exclusion of individual studies. The recalculated estimates ranged from 9% to 17%, compared with 11% in the complete GLMM. Exclusion of Demirci et al. (2015) [23], which reported no colour-alteration events, produced the highest pooled estimate of 17% (95% CI: 10–27%). Conversely, exclusion of Korkut et al. (2021) [9], which reported the highest number of events, resulted in the lowest estimate of 9% (95% CI: 3–23%). All leave-one-out confidence intervals overlapped the estimate obtained from the complete model, and the exclusion of no individual study materially changed the overall clinical interpretation. These findings indicate that the conclusion of generally favourable long-term colour performance was not dependent on any single study (Figure 6).

### 3.6. Influence and Outlier Analysis of Colour Alteration

**Figure 7:**
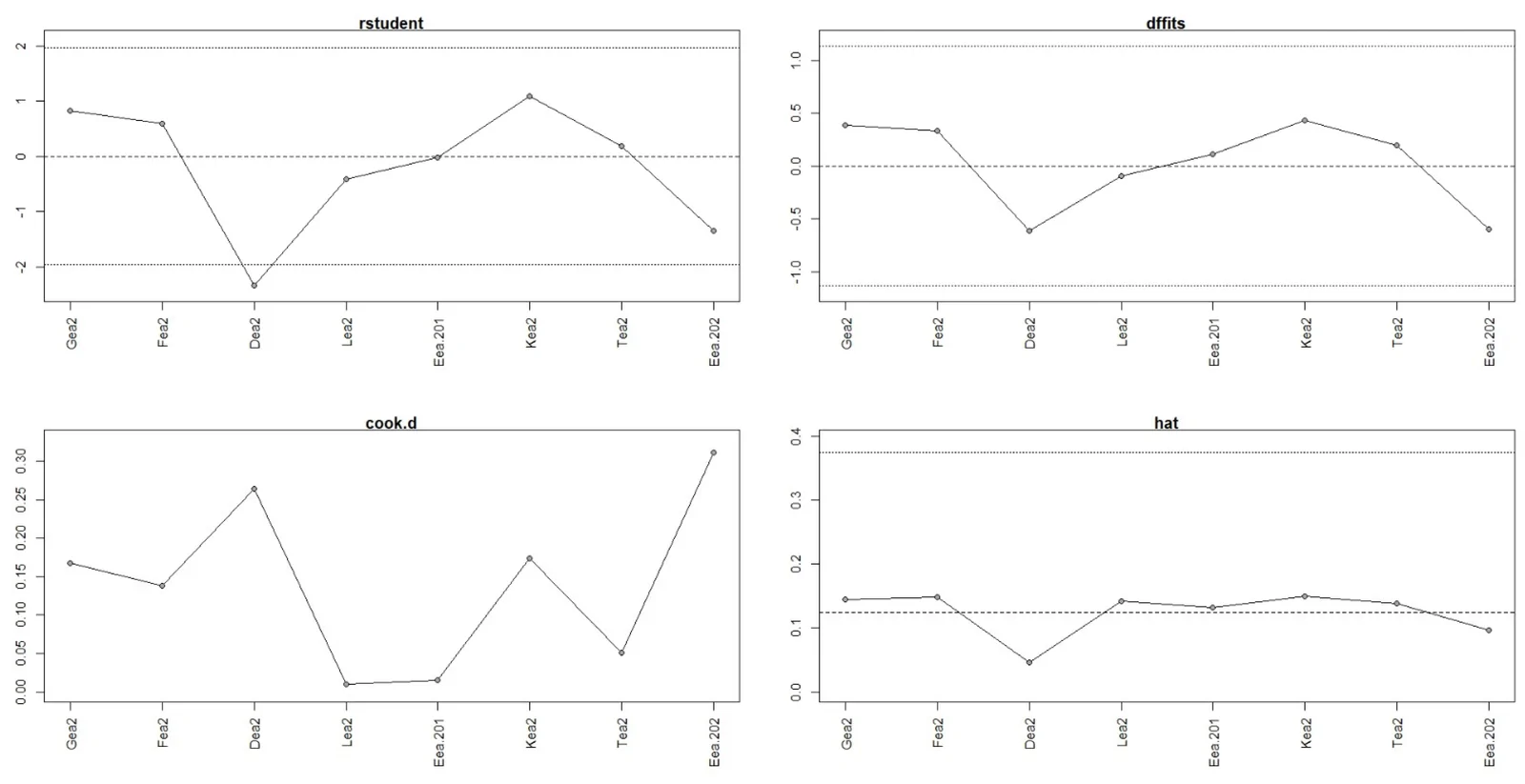
Influence and outlier diagnostics for the meta-analysis of colour alteration in freehand direct anterior composite resin restorations. The panels present externally studentized residuals, DFFITS values, Cook’s distances, and leverage values; horizontal reference lines indicate the corresponding diagnostic thresholds.

Influence diagnostics indicated that no individual study exerted a disproportionate influence on the pooled proportion of colour alteration. Demirci et al. (2015) [23] exhibited an externally studentized residual below −1.96 and was therefore identified as a potential low outlier according to the conventional criterion. However, this residual did not exceed the more conservative Bonferroni-adjusted threshold, and the study was not classified as influential by the combined diagnostic criteria. Although Elkaffas et al. (2024) [27] showed the largest Cook’s distance, its value remained below the corresponding influence threshold. Similarly, all DFFITS and leverage values were below their predefined cut-offs. Overall, the analysis identified no influential study capable of materially distorting the pooled estimate, supporting the robustness of the findings for long-term colour alteration (Figure 7).

### 3.7. Pooled Proportion of Marginal Discolouration

Six independent studies comprising 566 freehand direct anterior composite resin restorations were included in the quantitative synthesis of marginal discolouration. Using a random-effects model with Freeman–Tukey double-arcsine transformation, the pooled proportion of restorations presenting marginal discolouration was 21% (95% CI: 10–34%). Equivalently, approximately 79% of the restorations were free from recorded marginal discolouration at the final eligible assessment, supporting generally favourable long-term marginal aesthetic performance. Individual study estimates ranged from 5% to 39%. Study weights were relatively balanced, ranging from 16.1% to 17.4%, indicating that no individual study disproportionately influenced the pooled estimate.

Substantial between-study heterogeneity was identified (τ^2^ = 0.0305; Cochran’s Q = 60.81, df = 5, *p* < 0.0001; I^2^ = 91.8%). The corresponding 95% prediction interval ranged from 0% to 68%, suggesting that the proportion of marginal discolouration reported by a future comparable study could vary markedly. This variability may be related to differences in composite formulations, adhesive and restorative procedures, finishing and polishing protocols, evaluation criteria, follow-up duration, operator-related factors, and patient exposure to dietary or behavioural staining agents.

**Figure 8:**
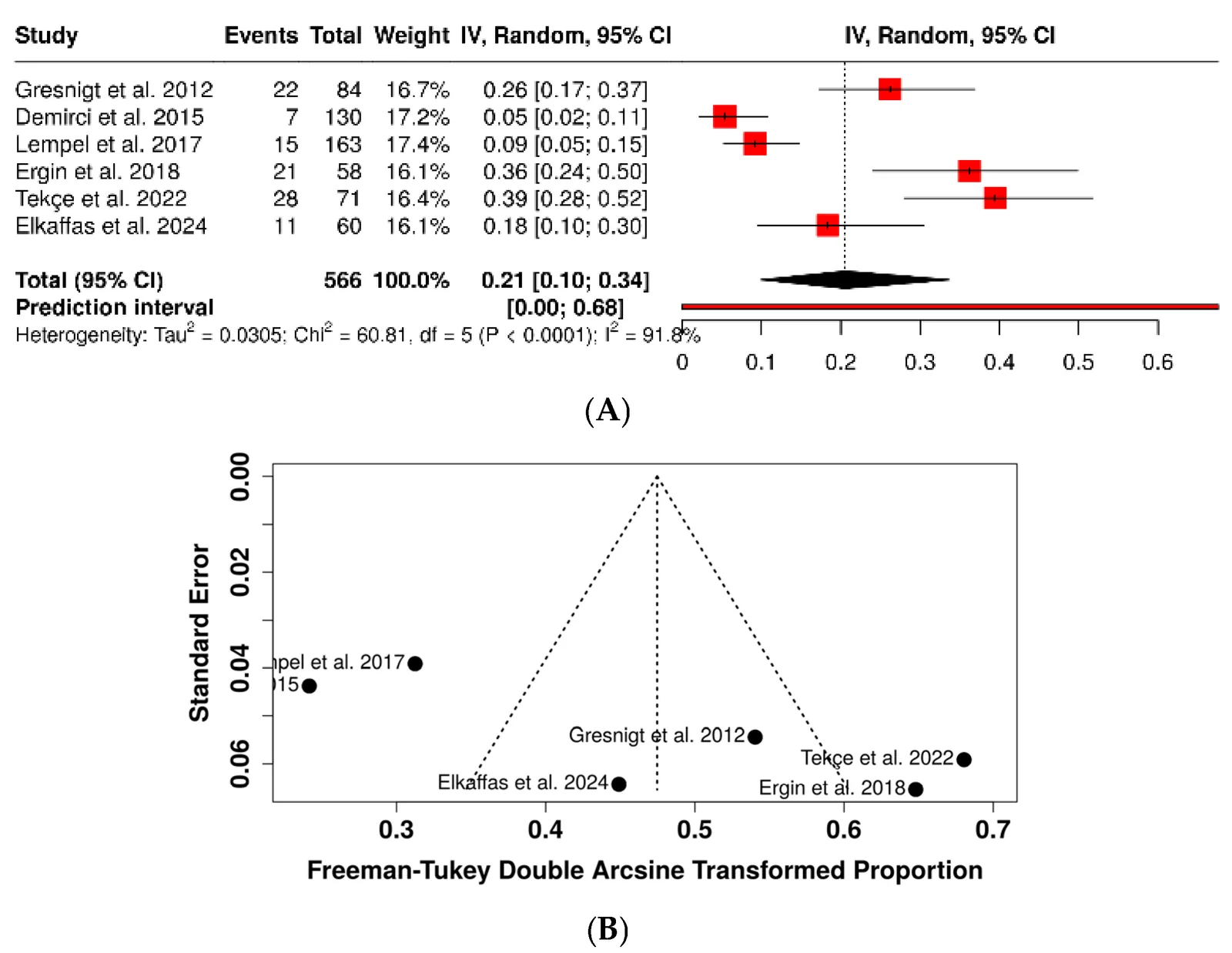
Meta-analysis of marginal discolouration in freehand direct anterior composite resin restorations. (**A**) Forest plot showing the pooled proportion of marginal discolouration obtained using a random-effects model with Freeman–Tukey double-arcsine transformation. (**B**) Funnel plot illustrating the distribution of study estimates and potential small-study effects [8,23,24,25,26,27].

Visual inspection of the funnel plot revealed considerable dispersion and an apparently asymmetric distribution of the study estimates around the pooled effect. Studies reporting lower proportions of marginal discolouration were predominantly located on the left side of the plot, whereas estimates representing higher proportions were distributed towards the right. Nevertheless, only six studies were available, and substantial heterogeneity was present. The apparent asymmetry may therefore reflect genuine clinical and methodological variability rather than publication bias. Accordingly, the funnel plot was interpreted as exploratory, and publication bias or small-study effects could neither be confirmed nor excluded (Figure 8).

### 3.8. Sensitivity Analysis of Marginal Discolouration

**Figure 9:**
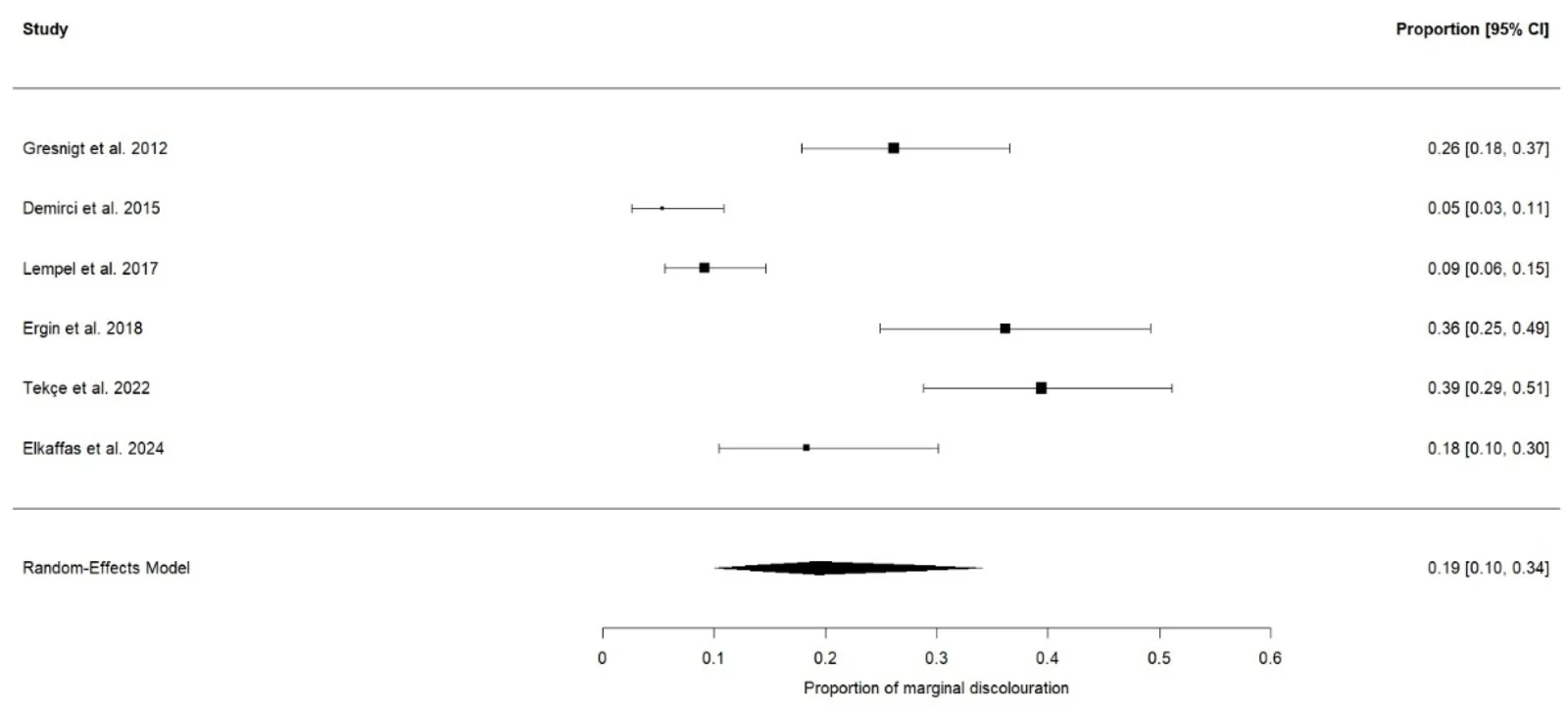
Forest plot of the pooled proportion of marginal discolouration in freehand direct anterior composite resin restorations using a logit-transformed random-effects model with REML estimation [8,23,24,25,26,27].

Six independent studies comprising 566 freehand direct anterior composite resin restorations were included in the sensitivity analysis. Using a random-effects model based on logit-transformed proportions and restricted maximum likelihood estimation, the pooled proportion of restorations presenting marginal discolouration was 19% (95% CI: 10–34%). Accordingly, approximately 81% of the restorations were free from recorded marginal discolouration at the final eligible assessment, indicating generally favourable long-term marginal aesthetic performance. Individual study estimates ranged from 5% to 39%, demonstrating variability among the evaluated clinical settings. The pooled estimate was consistent with the primary Freeman–Tukey analysis, which yielded a proportion of 21% (95% CI: 10–34%). This close agreement supports the robustness of the findings regarding marginal discolouration (Figure 9).

### 3.9. GLMM Analysis of Marginal Discolouration

**Figure 10:**
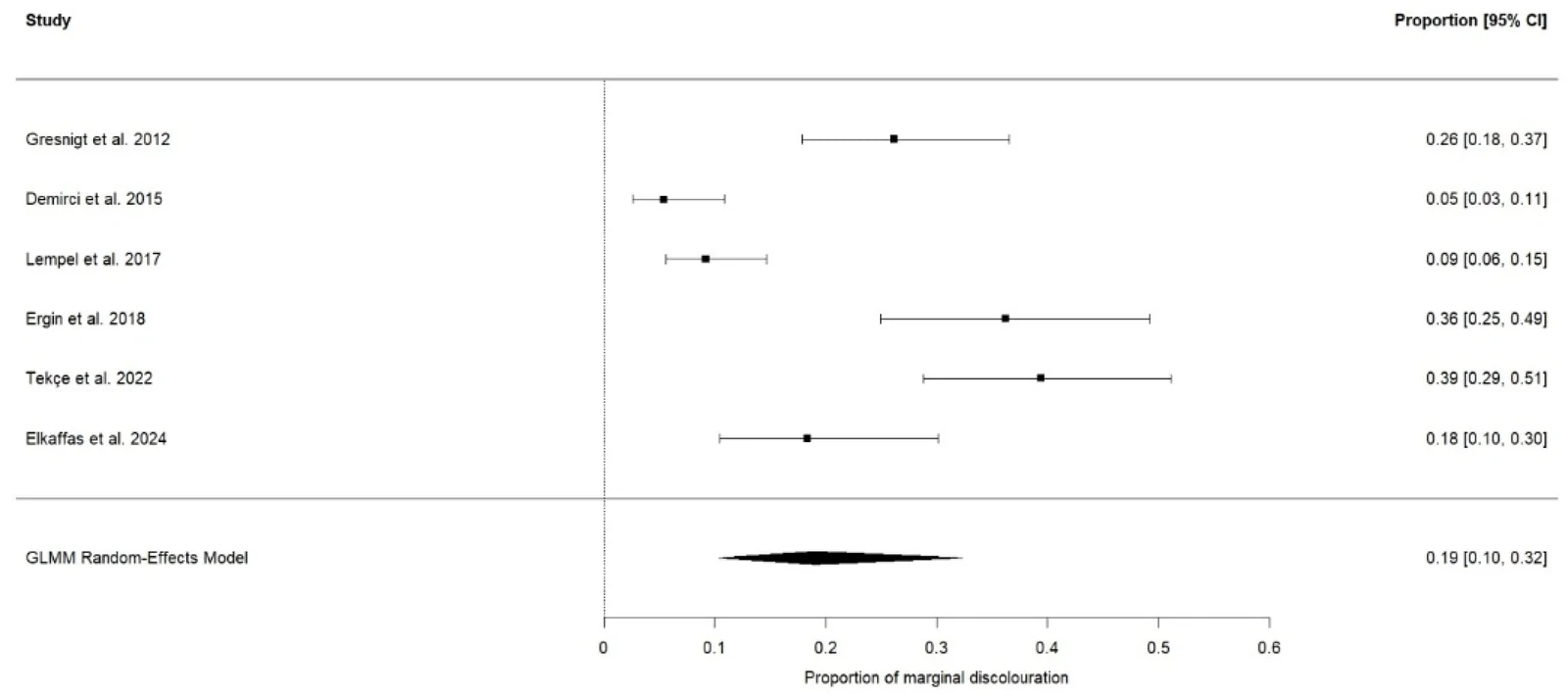
Forest plot of the pooled proportion of marginal discolouration in freehand direct anterior composite resin restorations using a binomial-normal generalized linear mixed model [8,23,24,25,26,27].

Six independent studies comprising 566 freehand direct anterior composite resin restorations were included in the generalized linear mixed-model analysis. The binomial-normal GLMM estimated a pooled proportion of marginal discolouration of 19% (95% CI: 10–32%). Accordingly, approximately 81% of the restorations were free from recorded marginal discolouration at the final eligible assessment, indicating generally favourable long-term marginal aesthetic performance. Individual study estimates ranged from 5% to 39%, demonstrating variability across the included clinical settings. The GLMM estimate was consistent with those obtained using the Freeman–Tukey model (21%; 95% CI: 10–34%) and the logit-REML model (19%; 95% CI: 10–34%). The close agreement among the three analytical approaches supports the robustness of the pooled result (Figure 10).

### 3.10. Hartung–Knapp Sensitivity Analysis of Marginal Discolouration

**Figure 11:**
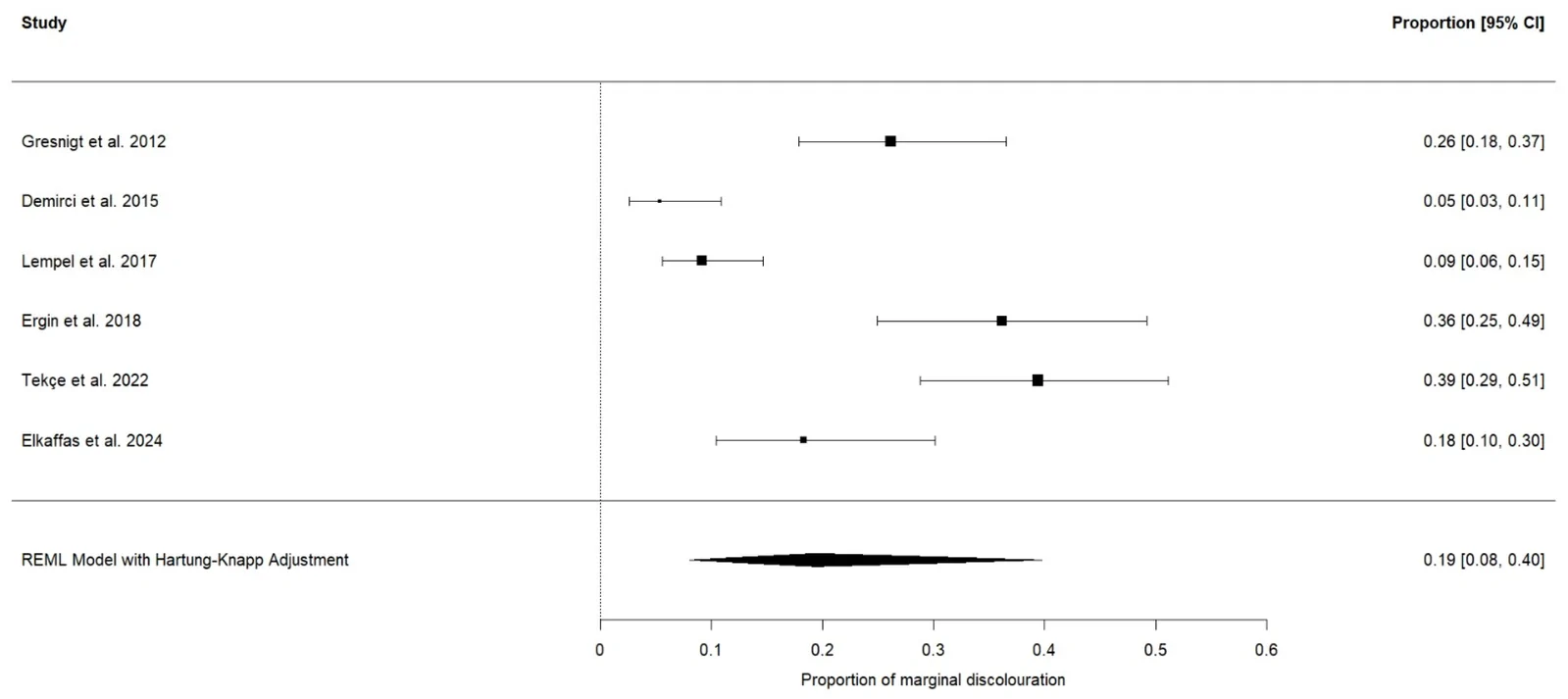
Forest plot of the pooled proportion of marginal discolouration in freehand direct anterior composite resin restorations using a REML random-effects model with Hartung–Knapp adjustment [8,23,24,25,26,27].

Six independent studies comprising 566 freehand direct anterior composite resin restorations were included in the Hartung–Knapp sensitivity analysis. The REML random-effects model with Hartung–Knapp adjustment yielded a pooled proportion of marginal discolouration of 19% (95% CI: 8–40%). Accordingly, approximately 81% of the restorations were free from recorded marginal discolouration at the final eligible assessment, supporting generally favourable long-term marginal aesthetic performance. The pooled estimate remained unchanged from the conventional logit-REML model; however, the confidence interval widened from 10–34% to 8–40%, reflecting the additional uncertainty associated with the small number of available studies. The result was also compatible with the GLMM estimate of 19% (95% CI: 10–32%). Overall, the Hartung–Knapp analysis preserved the direction and magnitude of the primary finding while demonstrating greater uncertainty around the precise pooled proportion (Figure 11).

### 3.11. Leave-One-Out Sensitivity Analysis of Marginal Discolouration

**Figure 12:**
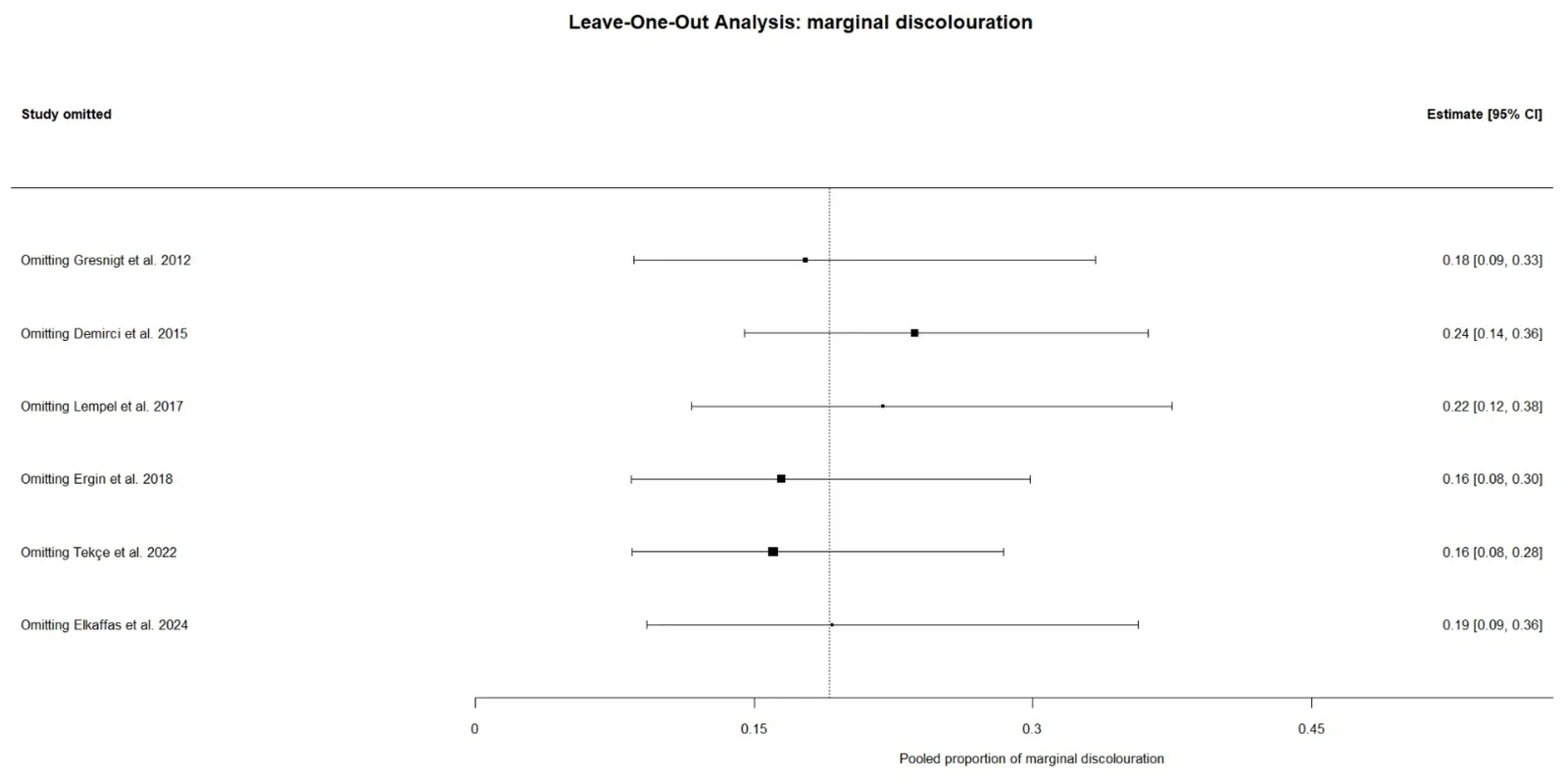
Leave-one-out sensitivity analysis of the pooled proportion of marginal discolouration in freehand direct anterior composite resin restorations. The vertical dotted line represents the estimate obtained from the complete GLMM [8,23,24,25,26,27].

The leave-one-out sensitivity analysis demonstrated that the pooled proportion of marginal discolouration was robust to the sequential exclusion of individual studies. The recalculated estimates ranged from 16% to 24%, compared with 19% in the complete GLMM. Exclusion of Demirci et al. (2015) [23], which reported one of the lowest proportions of marginal discolouration, resulted in the highest pooled estimate of 24% (95% CI: 14–36%). Conversely, exclusion of Ergin et al. (2018) [25] or Tekçe et al. (2022) [26] yielded the lowest pooled estimate of 16%, with 95% confidence intervals of 8–30% and 8–28%, respectively. All leave-one-out confidence intervals overlapped the complete-model estimate, and the exclusion of no individual study materially altered the overall clinical interpretation. These findings indicate that the estimated long-term marginal aesthetic performance was not driven by any single study (Figure 12).

### 3.12. Influence and Outlier Analysis of Marginal Discolouration

**Figure 13:**
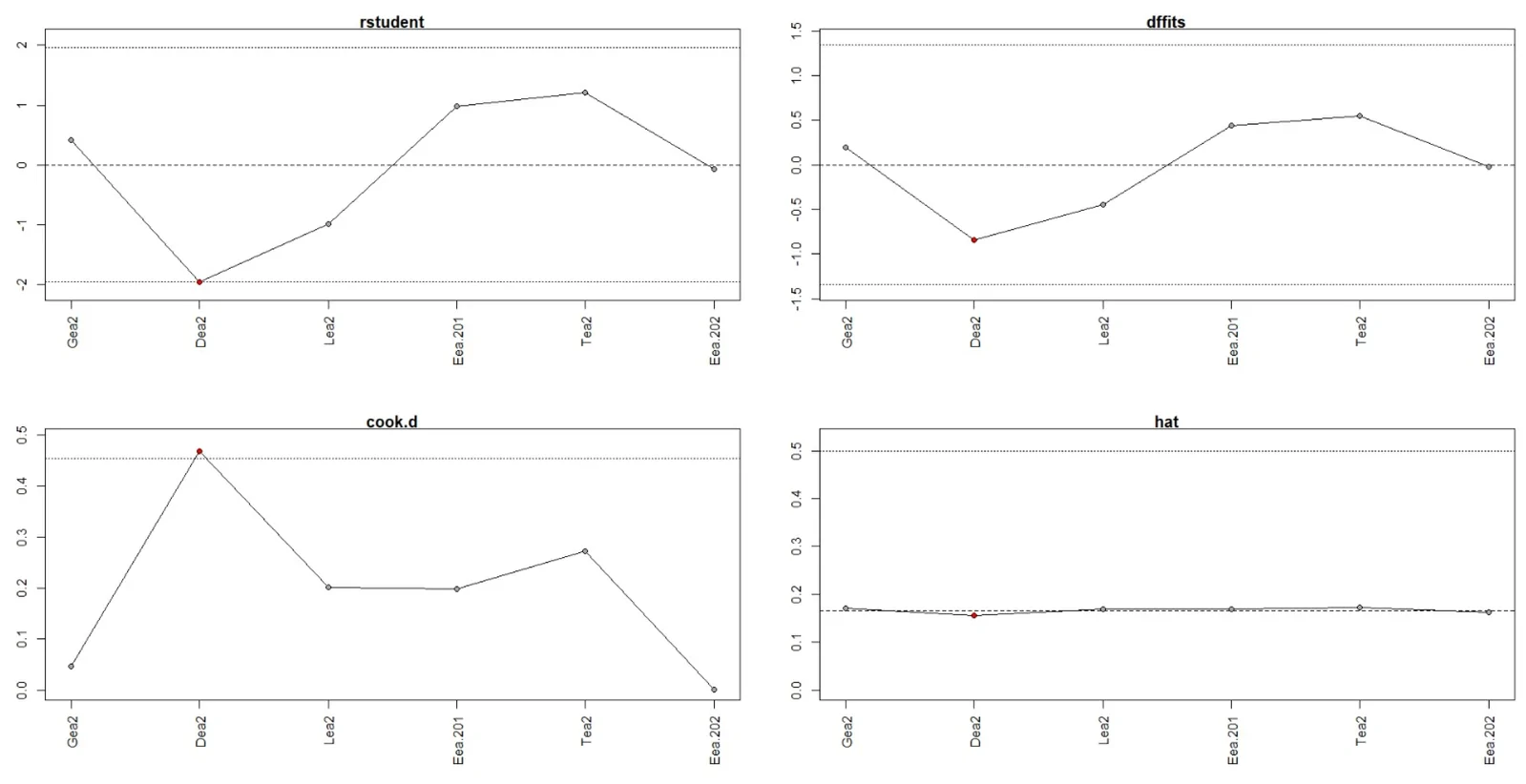
Influence and outlier diagnostics for the meta-analysis of marginal discolouration in freehand direct anterior composite resin restorations. The panels present externally studentized residuals, DFFITS values, Cook’s distances, and leverage values; highlighted points indicate studies classified as potentially influential.

Influence diagnostics identified Demirci et al. (2015) [23] as a potentially influential study in the meta-analysis of marginal discolouration. This study exhibited an externally studentized residual close to −1.96 and a Cook’s distance slightly exceeding the corresponding diagnostic threshold. However, its residual did not exceed the more conservative Bonferroni-adjusted cut-off, while its DFFITS and leverage values remained below their respective thresholds. No other study was identified as potentially influential or atypical. Consistent with these findings, the leave-one-out analysis showed that exclusion of Demirci et al. increased the pooled proportion only moderately, from 19% to 24% (95% CI: 14–36%), without changing the overall clinical interpretation. Therefore, although Demirci et al. contributed to the variability of the pooled estimate, the principal finding remained robust, and there was no statistical justification for excluding the study from the primary analysis (Figure 13).

### 3.13. Pooled Proportion of Surface-Quality Deterioration

Eight independent studies comprising 896 freehand direct anterior composite resin restorations were included in the quantitative synthesis of surface-quality deterioration. Using a random-effects model with Freeman–Tukey double-arcsine transformation, the pooled proportion of restorations presenting surface-quality deterioration was 21% (95% CI: 9–35%). Equivalently, approximately 79% of the restorations were free from recorded surface-quality deterioration at the final eligible assessment, indicating generally favourable long-term preservation of their surface characteristics. Individual study estimates ranged from 2% to 42%. The study weights were evenly distributed, ranging from 12.1% to 12.8%, indicating that no individual study disproportionately influenced the pooled estimate.

Considerable between-study heterogeneity was observed (τ^2^ = 0.0498; Cochran’s Q = 160.09, df = 7, *p* < 0.0001; I^2^ = 95.6%). The 95% prediction interval ranged from 0% to 74%, demonstrating that the proportion of surface-quality deterioration expected in a future comparable study could vary substantially. Such variability may be attributable to differences in composite formulation, finishing and polishing procedures, outcome definitions, clinical evaluation systems, follow-up duration, operator technique, oral hygiene, and patient-related exposure to mechanical and chemical challenges.

**Figure 14:**
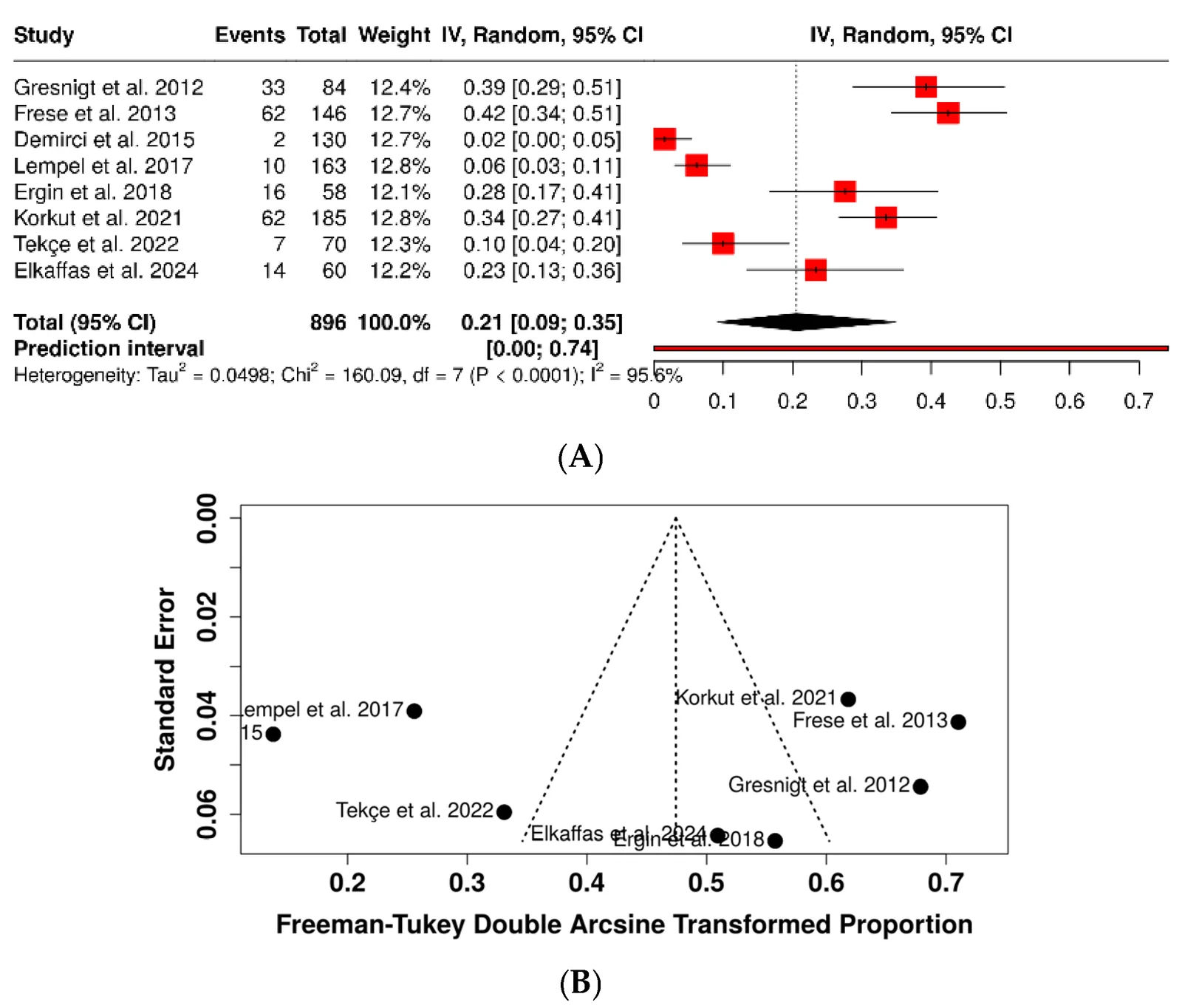
Meta-analysis of surface-quality deterioration in freehand direct anterior composite resin restorations. (**A**) Forest plot showing the pooled proportion of surface-quality deterioration obtained using a random-effects model with Freeman–Tukey double-arcsine transformation. (**B**) Funnel plot illustrating the distribution of study estimates and potential small-study effects [8,9,22,23,24,25,26,27].

Visual inspection of the funnel plot revealed considerable dispersion and an apparently asymmetric distribution of the study estimates around the pooled effect. Studies reporting both very low and relatively high proportions of surface-quality deterioration were observed, with several estimates located outside the pseudo-95% confidence limits. However, the interpretation of this pattern is limited by the small number of included studies and the substantial heterogeneity. The apparent asymmetry may therefore reflect genuine clinical and methodological variability rather than publication bias or small-study effects. Accordingly, the funnel plot was regarded as exploratory, and publication bias could neither be confirmed nor excluded (Figure 14).

### 3.14. Sensitivity Analysis of Surface-Quality Deterioration

**Figure 15:**
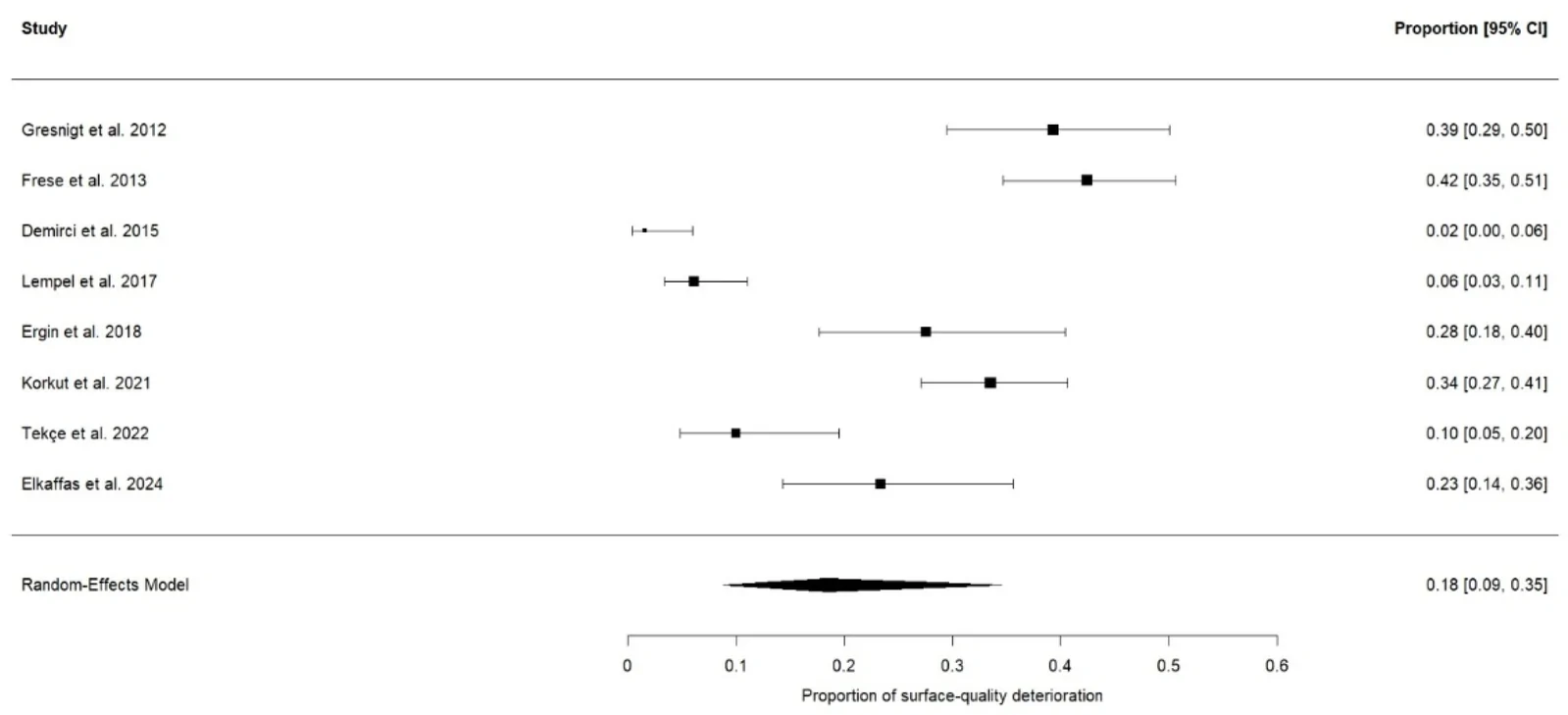
Forest plot of the pooled proportion of surface-quality deterioration in freehand direct anterior composite resin restorations using a logit-transformed random-effects model with REML estimation [8,9,22,23,24,25,26,27].

Eight independent studies comprising 896 freehand direct anterior composite resin restorations were included in the sensitivity analysis. Using a random-effects model based on logit-transformed proportions and restricted maximum likelihood estimation, the pooled proportion of restorations presenting surface-quality deterioration was 18% (95% CI: 9–35%). Accordingly, approximately 82% of the restorations were free from recorded surface-quality deterioration at the final eligible assessment, indicating generally favourable long-term preservation of surface characteristics. Individual study estimates ranged from 2% to 42%, demonstrating considerable variability across clinical settings. The pooled estimate was consistent with the primary Freeman–Tukey analysis, which yielded a proportion of 21% (95% CI: 9–35%). The agreement between both analytical approaches supports the robustness of the overall finding (Figure 15).

### 3.15. GLMM Analysis of Surface-Quality Deterioration

**Figure 16:**
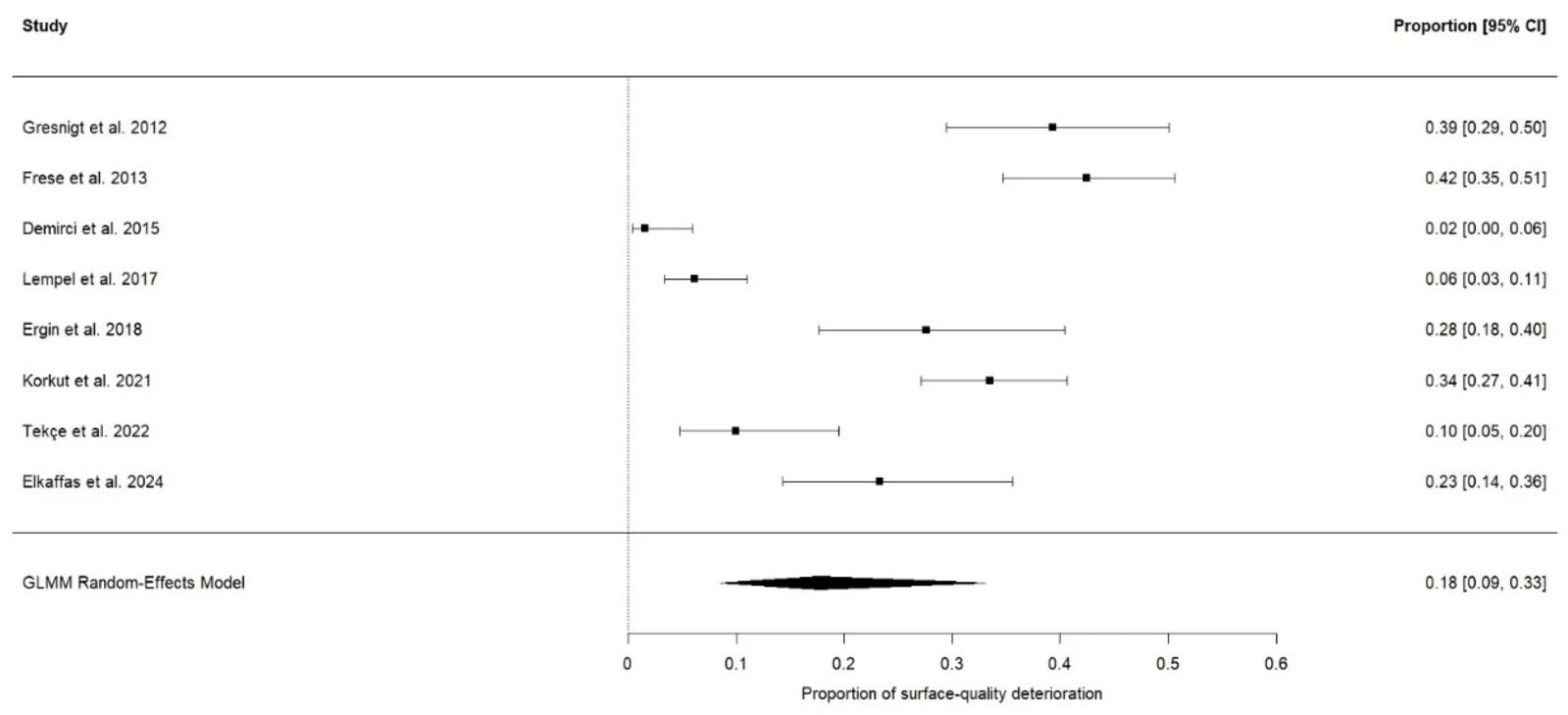
Forest plot of the pooled proportion of surface-quality deterioration in freehand direct anterior composite resin restorations using a binomial-normal generalized linear mixed model [8,9,22,23,24,25,26,27].

Eight independent studies comprising 896 freehand direct anterior composite resin restorations were included in the generalized linear mixed-model analysis. The binomial-normal GLMM estimated a pooled proportion of surface-quality deterioration of 18% (95% CI: 9–33%). Accordingly, approximately 82% of the restorations were free from recorded surface-quality deterioration at the final eligible assessment, indicating generally favourable long-term preservation of surface characteristics. Individual study estimates ranged from 2% to 42%, demonstrating substantial variability across the included clinical settings. The GLMM estimate was consistent with those obtained using the Freeman–Tukey model (21%; 95% CI: 9–35%) and the logit-REML model (18%; 95% CI: 9–35%). The close agreement among the three analytical approaches supports the robustness of the overall conclusion (Figure 16).

### 3.16. Hartung–Knapp Sensitivity Analysis of Surface-Quality Deterioration

**Figure 17:**
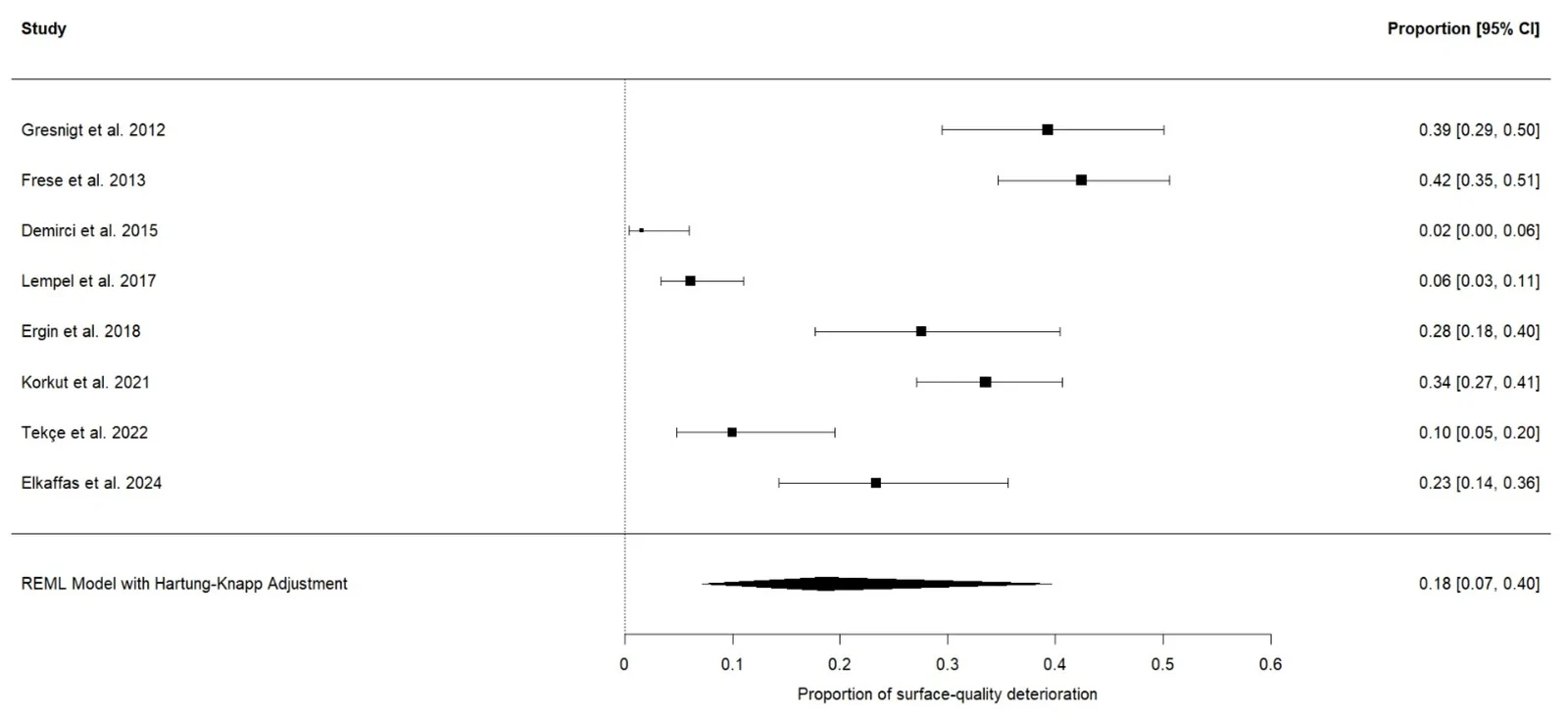
Forest plot of the pooled proportion of surface-quality deterioration in freehand direct anterior composite resin restorations using a REML random-effects model with Hartung–Knapp adjustment [8,9,22,23,24,25,26,27].

Eight independent studies comprising 896 freehand direct anterior composite resin restorations were included in the Hartung–Knapp sensitivity analysis. The REML random-effects model with Hartung–Knapp adjustment yielded a pooled proportion of surface-quality deterioration of 18% (95% CI: 7–40%). Accordingly, approximately 82% of the restorations were free from recorded surface-quality deterioration at the final eligible assessment, supporting generally favourable long-term preservation of surface characteristics. The pooled estimate remained unchanged from the conventional logit-REML model; however, the confidence interval widened from 9–35% to 7–40%, reflecting the additional uncertainty associated with the limited number of studies and the considerable variability among their estimates. The result was also compatible with the GLMM estimate of 18% (95% CI: 9–33%). Overall, the Hartung–Knapp analysis confirmed the stability of the pooled estimate while providing a more conservative representation of its uncertainty (Figure 17).

### 3.17. Leave-One-Out Sensitivity Analysis of Surface-Quality Deterioration

**Figure 18:**
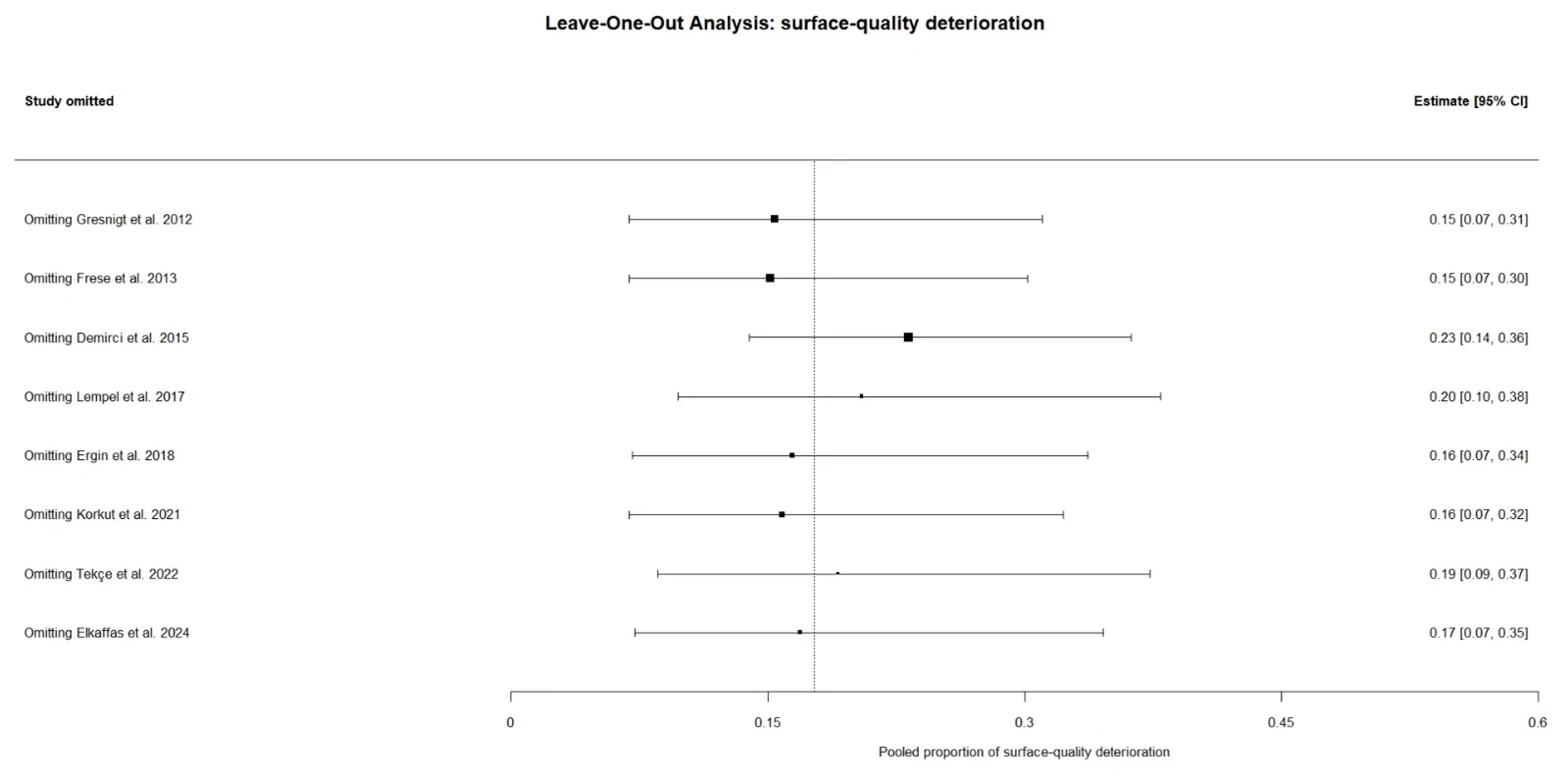
Leave-one-out sensitivity analysis of the pooled proportion of surface-quality deterioration in freehand direct anterior composite resin restorations. The vertical dotted line represents the estimate obtained from the complete GLMM [8,9,22,23,24,25,26,27].

The leave-one-out sensitivity analysis demonstrated that the pooled proportion of surface-quality deterioration remained stable after the sequential exclusion of individual studies. The recalculated estimates ranged from 15% to 23%, compared with 18% in the complete GLMM. Exclusion of Demirci et al. (2015) [23], which reported the lowest proportion of surface-quality deterioration, resulted in the highest pooled estimate of 23% (95% CI: 14–36%). Conversely, exclusion of Gresnigt et al. (2012) [8] or Frese et al. (2013) [22] yielded the lowest pooled estimate of 15%, with 95% confidence intervals of 7–31% and 7–30%, respectively. All leave-one-out confidence intervals overlapped the complete-model estimate, and no individual study materially altered the overall clinical interpretation. These findings support the robustness of the estimated long-term surface-quality performance (Figure 18).

### 3.18. Influence and Outlier Analysis of Surface-Quality Deterioration

**Figure 19:**
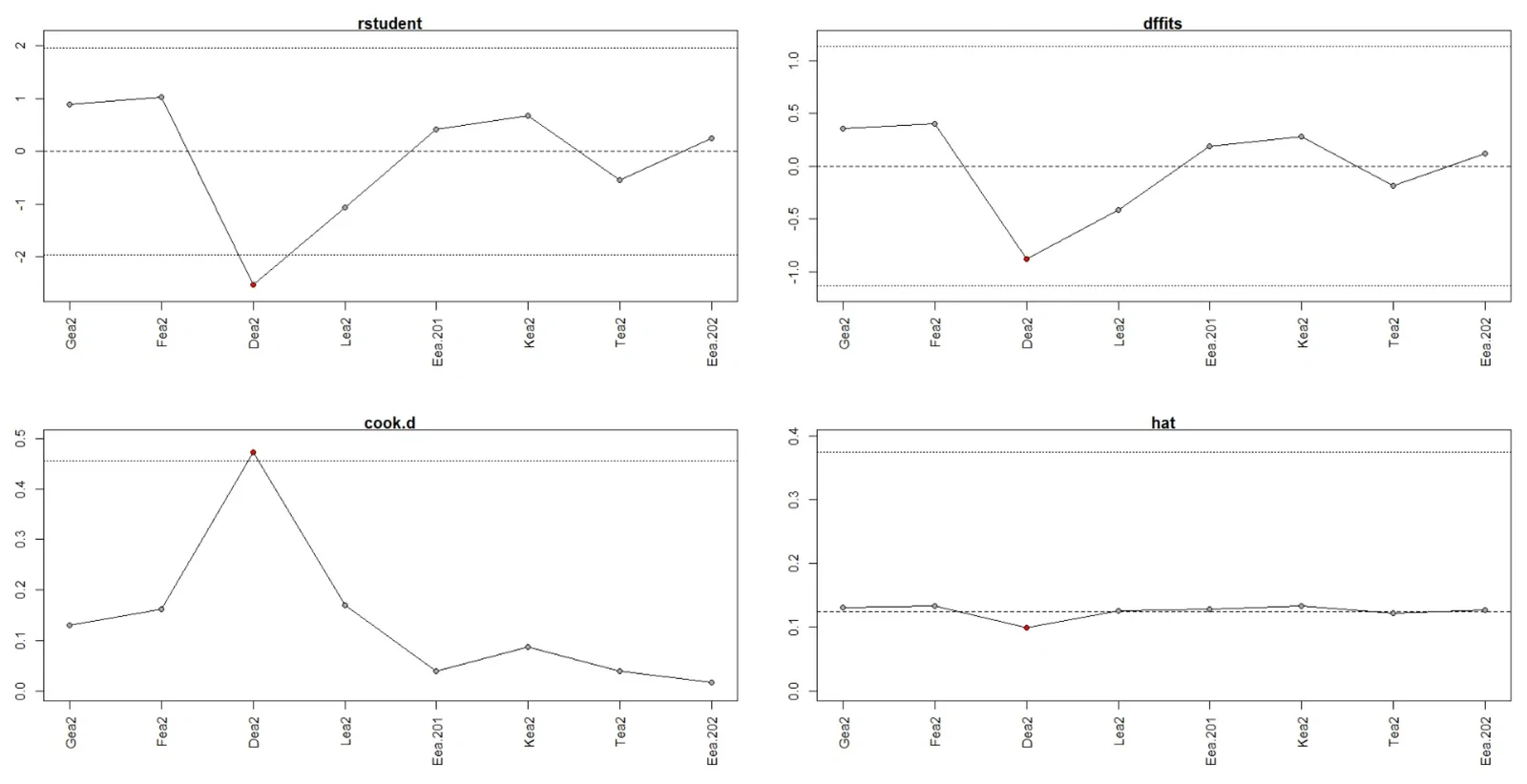
Influence and outlier diagnostics for the meta-analysis of surface-quality deterioration in freehand direct anterior composite resin restorations. The panels present externally studentized residuals, DFFITS values, Cook’s distances, and leverage values; highlighted points indicate studies classified as potentially influential.

Influence diagnostics identified Demirci et al. (2015) [23] as the only potentially influential study in the meta-analysis of surface-quality deterioration. This study presented an externally studentized residual of approximately −2.5, exceeding the conventional ±1.96 threshold and indicating a potentially atypically low proportion of surface deterioration. However, the residual did not exceed the more conservative Bonferroni-adjusted cut-off. Its Cook’s distance was slightly above the corresponding influence threshold, whereas its DFFITS and leverage values remained below their predefined limits. No other study showed evidence of being atypical or influential. Moreover, exclusion of Demirci et al. in the leave-one-out analysis increased the pooled proportion only moderately, from 18% to 23% (95% CI: 14–36%), without changing the overall clinical interpretation. Therefore, although this study contributed to between-study variability, the pooled finding remained robust, and its exclusion from the primary analysis was not statistically justified (Figure 19).

## 4. Discussion

To the best of our knowledge, this systematic review and meta-analysis is the first quantitative synthesis specifically designed to evaluate the long-term aesthetic performance of anterior direct composite restorations placed using a freehand technique, simultaneously examining colour alteration, marginal discolouration, and surface-quality deterioration. Unlike previous investigations focused primarily on restoration survival, this study evaluated aesthetic outcomes directly related to patient satisfaction and the clinical success of minimally invasive treatments. The findings showed that colour alteration occurred less frequently (14%) than marginal discolouration and surface-quality deterioration (21% for both outcomes), suggesting that aesthetic ageing is manifested mainly as localised changes at the tooth–restoration interface and on the restoration surface rather than as generalised colour alteration. Nevertheless, the substantial heterogeneity and wide prediction intervals indicate that these estimates represent average patterns rather than prognoses applicable to every clinical setting, because aesthetic performance depends on the interplay among material properties, restorative technique, clinical follow-up, and patient-related factors. Accordingly, the available evidence indicates that the longevity and aesthetic stability of anterior restorations are not determined solely by the biomaterial used but by a combination of clinical and biological variables that shape their long-term performance, underscoring the need to interpret these findings within the context of real-world clinical practice [3,4,5,30].

Marginal discolouration was among the most frequent aesthetic outcomes in this meta-analysis, with a pooled proportion of approximately 21%. However, this finding should not be interpreted as an indicator of restoration failure but rather as a manifestation of the progressive ageing of the adhesive interface. Hydrolytic degradation of the resin–adhesive complex, water sorption, surface wear, and pigment accumulation along the restoration margin are plausible mechanisms underlying this behaviour during clinical service. The substantial heterogeneity observed further suggests that the frequency of these changes is influenced more by differences in adhesive strategies, finishing and polishing protocols, follow-up duration, and patient-related factors than by the restorative material itself. This interpretation is consistent with the systematic review by Lim et al., who showed that direct composite veneers achieve high clinical survival rates despite the occurrence of minor aesthetic changes that can, in most cases, be managed using conservative procedures [6]. Similarly, Demarco et al. noted that the longevity of adhesive restorations depends on the interaction among the restorative material, operative technique, clinician experience, and patient-related factors, whereas Coelho-de-Souza et al. found that many localised aesthetic changes can be corrected through refurbishment or repair without compromising restoration survival [7,12]. Taken together, these findings support a conservative clinical approach in which marginal discolouration is regarded as an age-related restorative change rather than an immediate indication for restoration replacement.

Surface-quality deterioration occurred at a frequency similar to that of marginal discolouration, confirming that surface changes are among the principal manifestations of clinical ageing in direct composite restorations. These changes result from physicochemical degradation of the organic matrix, functional wear, and mechanical fatigue—processes that increase surface roughness, reduce gloss, and promote pigment retention, thereby progressively compromising the restoration’s aesthetic integration. Nevertheless, the between-study variability indicates that the extent of this deterioration depends on multiple clinical factors, including restorative protocols, finishing and polishing techniques, material type, and follow-up duration. This interpretation is consistent with the findings of Gresnigt et al., who demonstrated that direct veneers can maintain favourable aesthetic outcomes when appropriate adhesive protocols are applied [8]. Similarly, Korkut and Türkmen observed that surface changes gradually increase over time without necessarily compromising the clinical performance of the restorations [9], whereas Peumans et al. documented adequate aesthetic stability after five years of follow-up despite the presence of mild surface changes [10]. Although Bilen and Türkun evaluated a restorative technique different from that included in this meta-analysis, their findings complement the available evidence by showing that these changes can remain within clinically acceptable limits when appropriate maintenance protocols are implemented [11]. Collectively, these findings support the view that anterior direct composite restorations should be regarded as conservative and repairable treatments whose long-term success depends not only on their mechanical properties but also on periodic clinical follow-up aimed at preserving surface quality and aesthetic integration.

The findings should also be interpreted in light of the methodological heterogeneity among the included studies. Differences in assessment criteria (USPHS, FDI, clinical evaluation, and instrumental colour measurements), together with the need to harmonise distinct classification systems, probably contributed to the observed between-study variability. Consequently, the pooled proportions should be interpreted as estimates of the frequency of detectable aesthetic changes rather than as the proportion of restorations requiring replacement. In this context, use of the CIEDE2000 formula represents an important methodological advance because it provides a more uniform and clinically representative assessment of colour differences than the conventional CIELAB system [13].

From a clinical perspective, the findings of this study reinforce the view that the success of anterior direct restorations should be assessed not only in terms of survival but also according to their ability to maintain acceptable aesthetic integration through appropriate finishing, polishing, and periodic maintenance protocols. Available evidence shows that these strategies significantly affect the surface roughness, gloss, and colour alteration of resin composites, thereby helping preserve their long-term aesthetic performance [14,15,16].

A major strength of this study is that, to the best of our knowledge, it is the first quantitative synthesis specifically focused on the aesthetic performance of freehand anterior direct restorations. It also incorporated different statistical approaches that support the consistency of the resulting estimates. Nevertheless, the findings should be interpreted in view of the small number of available studies and the heterogeneity in restorative protocols, follow-up periods, assessment methods, and methodological quality. Furthermore, limited reporting of patient-related factors and the small number of studies included in each meta-analysis constrain the generalisability of the conclusions and hinder the assessment of publication bias. Prospective clinical studies with extended follow-up, standardised assessment criteria, and homogeneous methodologies are therefore needed to increase the certainty of the available evidence [17].

Overall, the findings of this review indicate that anterior direct composite restorations exhibit favourable aesthetic performance over the medium and long term. Although marginal discolouration and surface-quality deterioration were more frequent than generalised colour alteration, in most cases these changes represent manifestations of clinical ageing that can be managed through conservative maintenance and repair procedures without necessarily compromising restorative success. Consequently, direct composite restorations remain a conservative, repairable, and clinically reliable therapeutic option for the aesthetic rehabilitation of the anterior dentition.

An additional limitation concerns the unit of analysis. Aesthetic outcomes were generally reported at the restoration level, while individual patients frequently contributed more than one restoration. Therefore, observations within a study may have been correlated rather than fully independent. Because patient-level data and intraclass correlation estimates were not consistently available, this clustering could not be incorporated into the meta-analysis. As a result, the uncertainty around some study-specific and pooled estimates may be underestimated, and the corresponding confidence intervals may be narrower than they would be under a fully cluster-adjusted analysis. Furthermore, given the substantial between-study heterogeneity and wide prediction intervals, the pooled proportions should be interpreted as average estimates across heterogeneous clinical settings rather than as uniform expected outcomes. Importantly, the absence of recorded deterioration should not be considered equivalent to overall aesthetic success or clinical acceptability.

Another limitation is the substantial variability in follow-up duration and clinical indications across the included studies. Final assessments ranged from approximately 2 years to as long as 5–10 years, and aesthetic deterioration is inherently time-dependent. Therefore, pooling the longest available follow-up from each study does not represent a common time point and may contribute to between-study variability. In addition, the included restorations encompassed different clinical indications and restorative configurations, including direct veneers, diastema closures, composite build-ups, aesthetic recontouring, and restorations placed in patients with amelogenesis imperfecta. Consequently, the pooled proportions should not be interpreted as applying uniformly to a single restorative indication, patient population, or follow-up duration.

An additional limitation is the substantial variability in follow-up duration across the included studies, ranging from 24 months to as long as 10 years. Because aesthetic deterioration is time-dependent, restorations assessed at markedly different follow-up periods may not have had comparable opportunities to develop aesthetic changes. Follow-up duration was not formally evaluated as a moderator because the limited number of studies precluded reliable subgroup or meta-regression analyses. Therefore, differences in follow-up time may have contributed to the observed between-study heterogeneity and should be considered when interpreting the pooled estimates.

Risk-of-bias assessments should also be considered when interpreting the pooled estimates. Four randomized clinical trials were judged as having some concerns, whereas one study was classified as having a high overall risk of bias, mainly because of concerns related to the randomization process, missing outcome data, and selective reporting. The observational studies also presented limitations regarding participant comparability, control of confounding, and incomplete follow-up. Importantly, the existing leave-one-out sensitivity analyses sequentially excluded each individual study, including the study judged to be at high risk of bias, and its exclusion did not materially alter the pooled estimates. Nevertheless, the presence of methodological concerns across several included studies reduces confidence in the precision and generalizability of the pooled findings.

Finally, the inclusion of only English-language full-text articles represents a potential source of language bias, as relevant evidence published in other languages may have been excluded. This restriction may limit the completeness and generalizability of the available evidence.

## 5. Conclusions

Within the limitations of the available evidence, the pooled proportions of recorded colour alteration, marginal discolouration, and surface-quality deterioration were 14%, 21%, and 21%, respectively, after at least 24 months of clinical service. However, substantial between-study heterogeneity and wide prediction intervals indicate marked variability across clinical settings and limit the generalizability of these pooled estimates. Accordingly, these proportions should be interpreted as descriptive average estimates across heterogeneous studies rather than as uniform expectations for individual patients or restorations. Importantly, the recorded events represent loss of ideal aesthetic status rather than clinical failure or clinical unacceptability, and the absence of recorded deterioration should not be considered equivalent to overall aesthetic success or clinical acceptability. Further well-designed prospective studies using standardized aesthetic criteria and consistent follow-up intervals are needed to improve the precision, comparability, and clinical applicability of the available evidence.

## Figures and Tables

**Table 1:** Clinical and methodological characteristics of the studies included in the systematic review and meta-analysis.

Author (Year)	Country	Study Design	Population and Clinical Indication	Restorative Material/Technique	Sample Size	Follow-Up	Assessment Criteria and Outcomes	Main Findings
Gresnigt et al. (2012) [8]	The Netherlands	Randomised split-mouth clinical trial	Adults requiring direct laminate veneers for discoloured, malformed, misaligned or previously restored anterior teeth	Enamel Plus HFO/Ena-Bond versus Miris2/Clearfil SE Bond; freehand multilayer veneers	23 patients; 96 restorations initially; 84 restorations evaluated for aesthetic outcomes at the final recall	Mean 41.3 months; range 25–45.7 months	Modified USPHS: colour match, marginal discolouration, surface roughness and other clinical outcomes	Overall survival was 87.5%. Survival did not differ significantly between materials. Surface roughness and marginal discolouration were the principal aesthetic deteriorations.
Frese et al. (2013) [22]	Germany	Retrospective longitudinal clinical study	Patients receiving direct composite build-ups for diastema closure and aesthetic tooth recontouring	Seven composite systems; predominantly Enamel Plus HFO, Artemis, Herculite XRV and EsthetX; direct build-up technique	58 patients; 176 restorations; 146 restorations included in the qualitative aesthetic evaluation	5 years	Modified USPHS/FDI: colour alteration, surface lustre, surface staining, anatomical form, and other clinical parameters	Five-year survival was 84.6%; all repaired restorations remained functional. Most restorations exhibited excellent or good clinical quality, although gradual deterioration of colour and surface lustre was observed.
Demirci et al. (2015) [23]	Turkey	Prospective randomised split-mouth clinical study	Patients requiring diastema closure or tooth recontouring, predominantly after orthodontic treatment	Filtek Supreme XT/Scotchbond Multi-Purpose versus CeramX Duo/XP Bond; direct composite build-ups	30 patients; 147 restorations initially; 130 available for the final aesthetic outcomes	4 years	Modified Ryge: colour match, marginal discolouration, surface texture, anatomical form, marginal adaptation and survival	Four-year survival was 92.8% for Filtek Supreme XT and 93.0% for CeramX Duo. No significant differences were found between materials; colour alteration and surface texture remained predominantly ideal.
Lempel et al. (2017) [24]	Hungary	Retrospective longitudinal cohort study	Adults with direct anterior composite build-ups for fractured teeth or diastema closure	Nanofill Filtek Supreme XT versus microhybrid Enamel Plus HFO; freehand multilayer build-ups	65 patients; 163 restorations; 160 evaluable for colour match	Mean 7.2 ± 1.4 years; range 5–10 years	Modified USPHS: colour match, marginal discolouration, surface roughness, fracture, and survival	Overall survival was 88.3%. Nanofill restorations showed significantly more colour mismatch, whereas microhybrid restorations experienced more fractures. Marginal and surface deterioration was generally limited.
Ergin et al. (2018) [25]	Turkey	Prospective comparative clinical study with randomised material allocation	Adults treated for maxillary anterior diastema closure and tooth reshaping	Filtek Z550/Adper Single Bond 2 versus Charisma Diamond/Gluma2 Bond; freehand composite build-ups	23 patients and 76 restorations initially; 19 patients and 58 restorations at 4 years	Mean 43.4 months; range 37–48 months	FDI criteria: colour match, surface lustre, surface and marginal staining, anatomical form, and survival	Survival was 96.3% for Filtek Z550 and 93.5% for Charisma Diamond. No significant between-material differences were identified; minor losses of lustre and marginal staining increased over time.
Korkut et al. (2021) [9]	Turkey	Retrospective longitudinal clinical study	Adults receiving maxillary anterior diastema closure or aesthetic recontouring restorations	Essentia Universal, Estelite Asteria and Ceram.x SphereTEC; monochromatic freehand layering without matrices or silicone indexes	53 patients; 216 restorations initially; 185 with extractable final aesthetic assessments	4 years	FDI criteria: colour match/translucency, surface lustre, combined surface/marginal staining, anatomical form, and survival	Overall survival was 90.3%, with no significant differences among materials. Essentia Universal showed more loss of lustre, staining, and colour mismatch than Estelite Asteria and Ceram.x SphereTEC.
Tekçe et al. (2022) [26]	Turkey	Prospective single-centre split-mouth clinical trial	Adolescents and adults with amelogenesis imperfecta treated with anterior direct laminate veneers	Nanohybrid Clearfil Majesty ES-2/Clearfil Universal Bond versus nanofill Filtek Ultimate/Single Bond Universal	15 patients; 91 restorations initially; 70–71 restorations with extractable 4-year aesthetic outcomes	4 years	Modified USPHS: colour match, marginal discolouration, surface texture, marginal adaptation and survival	Four-year success was 80.5% for the nanohybrid and 92.5% for the nanofill system. Differences were detected for marginal discolouration and surface texture after 3 years and for colour match after 4 years.
Elkaffas et al. (2024) [27]	Egypt	Double-blind parallel-group randomised clinical trial	Adults requiring multiple maxillary anterior diastema closures	Direct Estelite Asteria composite veneers versus indirect IPS e.max Press ceramic veneers; only the direct composite arm was used in the primary meta-analysis	28 patients; 120 veneers overall; 60 direct composite veneers	24 months	USPHS criteria: colour match, marginal discolouration, surface roughness and survival	Survival did not differ significantly between direct composite and ceramic veneers—93.4% versus 95.0%. Surface roughness and staining were more frequent in the direct composite group.

**Table 2:** Risk-of-bias assessment of randomized clinical trials using RoB 2.

Study	Randomization	Deviations from Intervention	Missing Outcome Data	Outcome Measurement	Selective Reporting	Overall
Gresnigt et al. (2012) [8]	Low	Low	Low	Low	Some concerns	Some concerns
Demirci et al. (2015) [23]	Low	Low	Low	Low	Some concerns	Some concerns
Ergin et al. (2018) [25]	Some concerns	Low	Some concerns	Low	Some concerns	High
Tekçe et al. (2022) [26]	Low	Low	Some concerns	Low	Some concerns	Some concerns
Elkaffas et al. (2024) [27]	Low	Low	Low	Low	Some concerns	Some concerns

**Table 3:** JBI critical appraisal of retrospective cohort studies. Y: Yes; N: No; U: Unclear.

Study	J1	J2	J3	J4	J5	J6	J7	J8	J9	J10	J11
Frese et al. (2013) [22]	U	Y	Y	Y	N	U	Y	Y	N	N	Y
Lempel et al. (2017) [24]	Y	Y	Y	Y	Y	U	Y	Y	N	N	Y
Korkut et al. (2021) [9]	U	Y	Y	Y	Y	Y	Y	Y	Y	Y	Y

## Data Availability

The raw data supporting the conclusions of this article will be made available by the authors, without undue reservation.

## Supplementary Materials

The following supporting information can be downloaded at: https://www.mdpi.com/article/10.3390/dj14100633/s1, Table S1: PRISMA 2020 checklist; Table S2: Study-level outcome data and harmonization of clinical ratings used to define aesthetic deterioration.
