# Supplementary material for: Long-Term Aesthetic Performance of Freehand Direct Anterior Composite Resin Restorations: A Systematic Review and Meta-Analysis of Colour Alteration, Marginal Discolouration, and Surface-Quality Deterioration
Source: Dent J (Basel). 2026 Oct 2;14(10):633. doi: 10.3390/dj14100633 (PMC13702972; doi:10.3390/dj14100633)
Supplement: Supplementary file 1 [file dentistry-14-00633-s001.zip › dentistry-4527237-supplementary-Table S2.pdf]

Table S2. Study-level outcome data and harmonization of clinical ratings used to define aesthetic deterioration.

Study	Assessment system	Classification used in the meta-analysis	Colour alteration, events/N	Marginal discolouration, events/N	Surface-quality deterioration, events/N
Gresnigt et al. (2012)	Modified USPHS	Ideal aesthetic category = non-event; any deterioration from ideal = event	25/84	22/84	33/84
Frese et al. (2013)	Modified USPHS/FDI	Ideal aesthetic category = non-event; any deterioration from ideal = event	36/146	—	62/146
Demirci et al. (2015)	Modified Ryge	Ideal aesthetic category = non-event; any deterioration from ideal = event	0/130	7/130	2/130
Lempel et al. (2017)	Modified USPHS	Ideal aesthetic category = non-event; any deterioration from ideal = event	15/160	15/163	10/163
Ergin et al. (2018)	FDI	Ideal aesthetic category = non-event; any deterioration from ideal = event	8/58	21/58	16/58

Korkut et al. (2021)	FDI	Ideal aesthetic category = non-event; any deterioration from ideal = event	64/185	—	62/185
Tekçe et al. (2022)	Modified USPHS	Ideal aesthetic category = non-event; any deterioration from ideal = event	12/70	28/71	7/70
Elkaffas et al. (2024)	USPHS	Ideal aesthetic category = non-event; any deterioration from ideal = event	2/60	11/60	14/60

— indicates that the outcome was not reported separately or was not extractable for the corresponding meta-analysis
